# Sortilin exhibits tumor suppressor-like activity by limiting EGFR transduction function

**DOI:** 10.1038/s41388-026-03680-5

**Published:** 2026-02-14

**Authors:** E. Lapeyronnie, C. Granet, J. Tricard, F. Gallet, M. Yassine, H. Daverat, A. Rovini, A. Chermat, MO Jauberteau, F. Bertin, B. Melloni, F. Vincent, T. Naves, F. Lalloué

**Affiliations:** 1 UMR Inserm 1308 CAPTuR, Contrôle de l’Activation cellulaire, Progression Tumorale et Résistance thérapeutique and Chaire de Pneumologie Expérimentale, Université de Limoges, Faculté de Médecined, Limoges, CEDEX France; 2 Service de Chirurgie Thoracique et Cardio-vasculaire, Centre Hospitalier et Universitaire de Limoges, Limoges, CEDEX France; 3 Service d’Immunologie Clinique, Centre Hospitalier et Universitaire de Limoges, Limoges, CEDEX France; 4 Service de Pathologie Respiratoire, Centre Hospitalier et Universitaire de Limoges, Limoges, CEDEX France

**Keywords:** Non-small-cell lung cancer, Mechanisms of disease

## Abstract

Lung cancer is the leading cause of cancer deaths worldwide and remains one of the most difficult to cure. Tyrosine kinase receptors, such as the epidermal growth factor receptor (EGFR), are often aberrantly activated by gene mutation and drive tumor growth. Monotherapy with tyrosine kinase inhibitors targeting EGFR has shown initial efficacy, but their benefits tend to decline over time. EGFR acts as a transcription factor promoting the expression of oncogenic drivers, which, in turn, cooperate with canonical EGFR mutations to induce therapeutic resistance. This study reports that sortilin, a crucial regulator of cytoplasmic EGFR, attenuates its transducing function. Genome-wide chromatin binding assays revealed that sortilin interacts with gene regulatory elements occupied by EGFR. These results suggest a model in which sortilin exhibits potential tumor suppressor-like activity by concurrently binding to regulatory elements of *cMYC*. Sortilin expression in lung adenocarcinoma may be predictive of the efficacy of anti-EGFR therapies.

## Introduction

Lung adenocarcinoma (LUAD), which represents more than 50% of patients with non-small cell lung cancer, remains the leading cause of cancer deaths worldwide [1]. About 15% of these tumors harbor somatic mutations in the gene encoding epidermal growth factor receptor (EGFR), constitutively activating the tyrosine kinase (TK) domain of EGFR, even in the absence of ligand stimulation. This sustained proliferative signaling [2] creates cells in which EGFR mutants act as principal oncogenic drivers [3]. Clinically, tyrosine kinase inhibitors (TKI) [4] limit the intensity and duration of EGFR proliferative signaling, thereby decreasing tumor aggressiveness and the course of disease [5]. However, although early and advanced LUAD do not differ in EGFR mutation frequency or type [6], the clinical benefits of TKIs decline over time [5, 7].

Irrespective of disease stage, co-oncogenic drivers cooperate with canonical EGFR mutations in maintaining tumor malignancy and enhancing relapse. Several studies have identified that EGFR translocation to the nucleus activates transcription factors [8–10], directly promoting the expression of these co-drivers, such as *cMYC*, which regulates the expression of genes activating cell cycle, epigenetic reprogramming, apoptosis, or *CCND1,* also implicated in cell proliferation [11–15]. These findings suggest that, independently of its TK activity, EGFR may be reoriented toward its nuclear signaling network, with the significance of its transcriptional functions potentially varying based on cancer type and stage [16, 17]. Thus, controlling the spatiotemporal distribution of EGFR remains crucial in limiting its oncogenic driving force. Several studies have already established a key role for sortilin, a sorting receptor belonging to the vacuolar protein sorting 10 (VSP10) family, in controlling EGFR protein levels through its internalization and degradation [18, 19]. Notably, we have previously reported that sortilin acts as a crucial regulator of EGFR endocytosis, limiting its proliferative signaling [18, 19]. To determine the putative clinical role of sortilin in the treatment of tumors with constitutively activated EGFR, this study investigated whether sortilin could also interfere with the nuclear EGFR signaling network and limit EGFR transcriptional program.

We have recently observed that EGFR–sortilin complexes are present in the nuclei of EGF-stimulated cells, concomitant with genome-wide chromatin binding. These complexes bind to transcription regulatory elements of genes associated with relapse from TKI treatment and disease progression [11, 20, 21]. Interestingly, sortilin preferentially binds to the transcription-starting site (TSS) of c*MYC*, subsequently reducing its activity. The TKI osimertinib triggers EGFR internalization and importation of EGFR–sortilin complexes into the cell nucleus, with sortilin expression in the nuclei repressing *cMYC* expression. Because sortilin expression is significantly lower than EGFR expression in LUAD cell lines, sortilin expression may act as a switch factor limiting EGFR transcriptional functions.

We have therefore proposed a model in which sortilin exhibits a potential tumor-suppressor-like activity by concurrently binding to the transcription regulatory elements of EGFR-targeted genes, thereby limiting the EGFR transcription activity. The present study provides new insights into the significance of sortilin expression in LUAD, especially in EGFR-positive tumors. Sortilin may both predict the efficacy of TKIs and be a new candidate for the treatment of LUAD.

## Materials and methods

### Chromatin immunoprecipitation (ChIP) assay

Chromatin immunoprecipitation (ChIP) assays were performed using SimpleChIP® Kits (Cell Signaling). DNA fragments were analyzed by agarose electrophoresis and quantified using a NanoDrop™ spectrophotometer. For immunoprecipitation, 50 µg DNA was mixed with ChIP buffer, protease inhibitor cocktail (PIC), and antibodies (EGFR H11, sortilin, normal Rabbit IgG, or mouse IgG1 control). After overnight incubation at 4 °C, ChIP-Grade Protein G Magnetic Beads were added, and the mixture was incubated for an additional 3 h. The beads were washed, and DNA-protein complexes were eluted, digested with proteinase K, and purified. ChIP-sequencing and bioinformatics analysis were performed by Novogene (Cambridge, UK).

### Subcellular fractionation

Nuclear and cytoplasmic fractions were extracted from cells using NE-PER™ Nuclear and Cytoplasmic Extraction Reagent kits (Thermo Scientific^TM^).

### Nuclear immunoprecipitation

Nuclear immunoprecipitations were performed with EGFR or sortilin antibodies and Protein G Dynabeads™. After adding nuclear lysates, the mixture was incubated at room temperature for 2 h. Beads were washed with PBS, and bound proteins were eluted with Laemmli buffer at 95 °C. Samples were analyzed by SDS-PAGE and western blotting.

### Immunoblotting

Cells were washed with ice-cold PBS and lysed with buffer containing SDS, 2-mercaptoethanol, glycerol, Tris-HCl, and protease inhibitors. Lysates were sonicated, and 30 µg protein aliquots were loaded onto SDS-PAGE gels for western blotting with specific antibodies (Supplementary Table 1). Blots were incubated with HRP-conjugated secondary antibodies and detected using enhanced chemiluminescence.

### Cell culture

The A549 cell line was isolated from a lung carcinomatous tissue with *KRAS*^G12V^ mutation and was obtained from the American Type Culture Collection (ATCC, ref. CCL-185). A549 pLKO cells represent empty vector control cells for A549 encoding short hairpin RNA (ShRNA) targeting either *EGFR* mRNA (ShEGFR) or *SORT1* mRNA (ShSORT1). Likewise, the H1975 cell line was isolated from a LUAD with *EGFR*^*L858R/T790M*^ mutation and was also obtained from the ATCC (ref. CRL-5908). H3255 cell line with EGFR ^*L858R*^ mutation was kindly provided by Sylvie Gazzeri of the Albert Bonniot Institute (France). HEK293T cells derived from human embryonic kidney cells (ATCC, ref. CRL-3216) are used for their high transfection efficiency. All cell lines were cultured in Dulbecco’s modified Eagle’s medium GlutaMAX (Gibco) supplemented with 10% fetal bovine serum (IDbio, France), 1% antibiotics (Gibco), and 1% non-essential amino acids (Gibco) at 37 °C in a humidified atmosphere containing 5% CO_2_. Where indicated, cells were either stimulated with 50 ng/mL EGF for 30 min, or treated with 1 µM of the TKI Osimertinib (AZD9291, Tagrisso, Cliniscience, France) according to Cross et al. [22] for 24 h. To ensure complete target inhibition and to maximize the detection of potential secondary effects or underlying mechanisms, a concentration of 1 µM osimertinib was used, despite being higher than the IC50.

### Generation of HEK293T knockout cell lines

CRISPR plasmids from Horizon (New England, UK), including sgRNA (Supplementary Table 2), Cas9, and Dasher GFP for single cell sorting, were used to generate knockout cell lines. HEK293T cells were transfected with CRISPR plasmids by using JetPEI (Ozyme) and cultured for 48 h. GFP-positive cells were sorted by fluorescence-activated cell sorting into 96-well plates to create isogenic clonal populations. These clones were expanded and screened respectively for EGFR or sortilin expression by western blotting to select HEK CRISPR^EGFR^ knockout or HEK CRISPR^SORT1^ knockout cells.

### Mice and in vivo tumor growth

Female NOD-SCID mice from Janvier Labs were housed in a pathogen-free environment. Experiments followed French Veterinary Department guidelines. H1975 cells overexpressing sortilin (induced by doxycycline) were engrafted into the mice left thighs. Tumor volume was measured twice a week. Mice received doxycycline (2 mg/mL) in drinking water after tumor development and were sacrificed 34 days post-engraftment. Tumors were collected for immunohistochemistry, qPCR, and western blotting.

### Immunofluorescence and confocal microscopy

Immunofluorescence and PLA (Proximity Ligation Assay) experiments were performed as previously described [18]. Quantitative analysis of each independent sample was performed using ImageJ software (NIH, Bethesda, Maryland, USA), based on the mean fluorescence values. At least 50 cells were analyzed per condition, with the results presented as a ratio relative to control cells. Fluorescent images were captured using epifluorescence microscopes (Zeiss Axiovert), equipped with a laser-scanning confocal imaging system (Zeiss LSM 510 META or LSM800).

### Plasmids, lentivirus-mediated RNA interference and siRNA interference

Cells were transiently transfected using JetPei (Polyplus Transfection) following the manufacturer’s instructions. Constructs for wild-type EGFR (EGFRwt) or the mutant EGFR^S229A^ impairing the nuclear translocation of EGFR were provided by Wei-Chien Huang [16]. Wild-type sortilin (full-length), which enables internalization following retrograde transport or a sortilin mutant lacking the intracellular C-terminal domain (sortilin Δc), was kindly provided by Gina Finan [23]. Sortilin Δc cannot be recycled from the plasma membrane. Inducible or constitutive sortilin-overexpressing cell lines were generated using custom lentivirus (AMSBIO, Milton, UK). For RNA interference assays, A549 cells were transfected with 100 nM siRNA targeting EGFR or control siRNA using INTERFERin (Polyplus Transfection).

### Total RNA extraction and quantitative (q-)PCR analysis

Total RNA was extracted from 50 mg of tissue or 1 million cells using QIAzol (QIAGEN). Aliquots of 2 µg RNA were reverse transcribed to cDNA with Superscript III (Invitrogen). Each qPCR reaction contained 50 ng cDNA, TaqMan probes, and Premix Ex Taq, and was run on a QuantStudio 3 (Applied Byosystem™) thermal cycler. RT-qPCR results were normalized to ACTB mRNA using the ΔΔCt method. ChIP-qPCR probes were synthesized by ThermoFisher Scientific (Supplementary Table 3).

### Patients and immunohistochemistry

Lung adenocarcinoma tumors, either frozen or embedded in paraffin blocks, were obtained from the Tumor Bank (Biolim) of Limoges University Hospital, following protocols approved by the Institutional Review Board (IRB) under the references AC-2013-1853 and DC-2011-1264 from the Anatomo-Pathology Department of CHU Dupuytren, Limoges. All patients were informed of the use of their tissue samples in research studies. Immunohistochemical and hematoxylin/eosin staining was performed on 5-μm-thick consecutive sections. Antibodies against sortilin (Alomone, Israel, 1/175, #ANT-009) and cMYC (Abcam, clone Y69, 1/200, ab32072) were used for tissue labeling on a Ventana Ultra at the Anatomo-Pathology department.

### Statistical analysis

Statistical analyses were performed using GraphPad Prism 9. Data are presented as the mean ± standard deviation from at least three independent experiments. ANOVA was used to determine statistical significance (*p* ≤ 0.05). Correlations between *cMYC* and *SORT1* mRNA levels in TCGA and CCLE databases were evaluated by linear regression with R software (version 3.6.1).

## Results

### Sortilin and EGFR nuclear interaction in EGF-stimulated cells

**Fig. 1:**
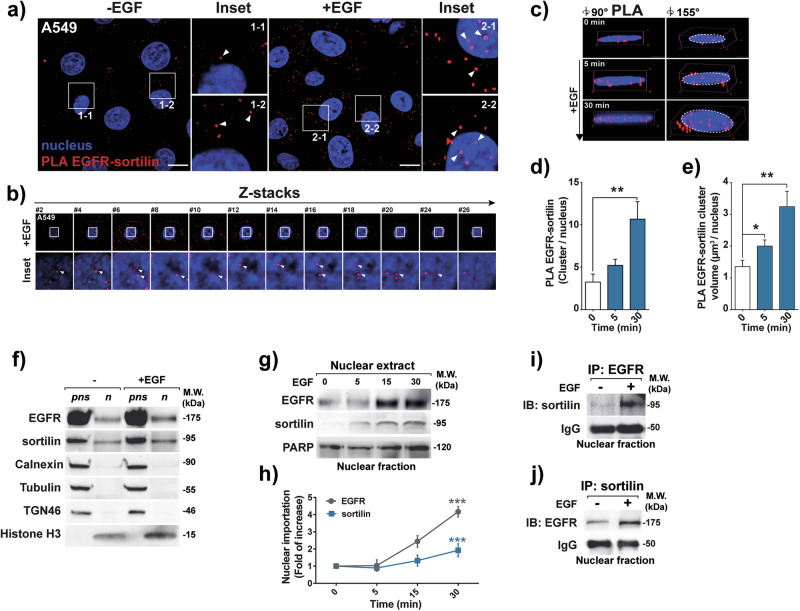
Sortilin and EGFR interact together in the nuclei of cancer cells. **a** Proximity ligation assay (PLA) showing the interaction between sortilin and EGFR in the lung adenocarcinoma cell line A549 in the absence (-EGF) or presence of EGF (+ EGF, 50 ng/mL) for 30 min. Red spots indicate sites of PLA amplification, reflecting interactions between sortilin and EGFR. Scale bar, 10 μm; white arrows show EGFR–sortilin clusters. **b** Z-stack sections of confocal microscopy images showing sortilin and EGFR interactions in z-axis (insets #2–26). White arrows show EGFR–sortilin clusters. **c** 3D confocal microscopy images showing EGFR–sortilin interactions at angles of 90° and 155° following EGF stimulation (0, 5 and 30 min with 50 ng/mL EGF). **d** Quantification of EGFR–sortilin spots per nucleus, following 0, 5 and 30 min of EGF stimulation (50 ng/mL). **e** Estimated volumes of EGFR–sortilin clusters per nucleus (µm^3^/nucleus following 0, 5 and 30 min of EGF stimulation (50 ng/mL)). **f** A549 cells were stimulated with EGF (+ EGF, 50 ng/mL) for 30 min or not, control (–), before solubilization to proceed with subcellular fractionation. The nuclear extract (n) and post-nuclear supernatant (pns) were then subjected to western blotting with anti-EGFR, anti-sortilin, and a set of antibodies directed against the endoplasmic reticulum with anti-calnexin, the cytoskeleton with anti-tubulin, the trans Golgi network with anti-TGN46 and the nucleus with anti-histone H3 to rule out cytoplasmic contamination in nuclear fraction. **g** Immunoblots showing kinetics of EGFR and sortilin nuclear importation following EGF stimulation of A549 cells. Nuclear fractions were obtained at 0, 5, 15, and 30 min after stimulation with 50 ng/mL EGF, PARP protein levels were used as a loading control. **h** Quantification of nuclear importation of EGFR and sortilin following EGF stimulation were normalized on PARP expression in fold of control. Molecular weights (MW) are shown in kilodaltons (kDa). **i**, **j** Confirmation of EGFR–sortilin interactions by nuclear co-immunoprecipitation of A549 cell lysates in the absence, control (–), or presence of EGF (+, 50 ng/mL) for 30 min and immunoblotted (IB) respectively with anti-EGFR or anti-sortilin antibodies.

Based on findings showing that sortilin limits EGFR proliferative signaling [18, 19], we tested whether sortilin exhibits a tumor suppressor-like activity by acting on its nuclear signaling network. Although sortilin physically interacts with EGFR at or near the plasma membrane in A549 cells, as previously reported [18, 19], we also detected EGFR–sortilin complexes toward the nuclei of cancer cells, following EGF stimulation, as shown by red spots indicating sites of proximity ligation amplification (PLA) (Fig. 1a; insets 1.1 to 2.2). Z-stack confocal images and three-dimensional projections at 90° and 155° confirmed that EGFR–sortilin complexes localized within the nuclei of EGF-stimulated cells (Fig. 1b; insets showing z-axis #2 to #26, and Fig. 1c), ruling out artificial superposition of red spots in the 2D plan. After 5 min of EGF stimulation, both the number of EGFR–sortilin clusters and their total nuclear volume increased significantly (*p* < *0.05*, Fig. 1c–e). This suggests that the translocation of EGFR–sortilin complexes begins early in the process of EGFR endocytosis.

To validate these findings, we isolated nuclei and post-nuclear supernatant (pns) fractions and performed western blotting to assess protein expression. We used specific markers for Trans Golgi Network (TGN46), endoplasmic reticulum (ER) (calnexin), and cytoskeleton (tubulin) to exclude cytoplasmic contamination in the nuclear fraction. Histone H3, a chromatin packaging protein, was used as a marker for the nuclear fraction. Our analysis revealed both EGFR and sortilin expression in the nuclei of EGF-stimulated cells (Fig. f), with expression increasing significantly at 30 min (*p* < *0.001*, Fig. 1g, h). These results corroborate confocal images, indicating translocation of EGFR and sortilin to the nuclei in response to EGF stimulation. Immunoprecipitation from isolated nuclei also demonstrated an increase in EGFR–sortilin complexes following EGF stimulation (Fig. 1i, j), further supporting the findings from PLA assay.

### Role of EGFR and sortilin in nuclear translocation

**Fig. 2:**
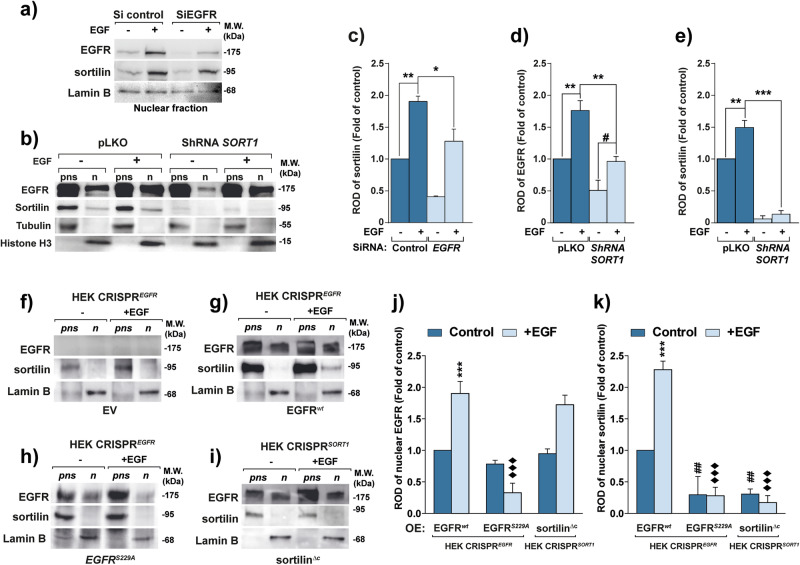
EGFR promotes sortilin nuclear translocation. **a** EGFR silencing by specific siRNA (SiEGFR) transfection for 72 h before assessment of sortilin importation into the nucleus by western blotting. **b** A549 with empty vector control (pLKO) and A549 sh*SORT1* cells were stimulated with EGF (+ EGF, 50 ng/mL) for 30 min or not (–) before subcellular fractionation. The nuclear extract (n) and post-nuclear supernatant (pns) were then subjected to western blotting with anti-EGFR, anti-sortilin, and antibodies directed against the cytoskeleton with anti-tubulin and the nucleus with anti-histone H3 to rule out cytoplasmic contamination in the nuclear fraction. **c** Relative optical density (ROD) of sortilin expression in isolated nuclei following EGFR depletion by siRNA. Histogram bars represent the relative optic density (ROD) of nuclear EGFR (**d**) or sortilin (**e**) normalized on histone H3 expression in fold of control in presence of EGF or not, following *SORT1* depletion (Sh*SORT1*). **f**–**i** HEK CRISPR cells for *EGFR* (HEK CRISPR^*EGFR*^) were respectively transfected with empty vector (EV), EGFR wild type (EGFR^*wt*^) or mutant EGFR S229A (EGFR^*S229A*^) while CRISPR *SORT1* cells (HEK CRISPR^*SORT1*^) were transfected with a C-tail truncated sortilin (sortilin^∆c^) mutant. Following 24 h of transfection and 30 min of EGF stimulation (+ EGF, 50 ng/mL) or not, control (–), we performed subcellular fractionation to isolated nucleus (n) from post-nuclear supernatant (pns). **j**–**k** Histogram bars represent the relative optic density (ROD) of nuclear EGFR or sortilin normalized on Lamin B expression in fold of control (respectively endogenous EGFR or sortilin) following different constructions upon EGF treatment (light blue bar) or not (dark blue bar). All values represent means ± SD. *p < 0.05, **p < 0.01, ***p < 0.001, ^#^*p* < 0.05, ^##^*p* < 0.01, ^###^*p* < 0.001, ^♦♦^*p*<0.05, ^♦♦^*p* < 0.01, ^♦♦♦^*p* < 0.001 by Student’s *t* test. Each experiment was repeated at least three times.

In light of these results, we next examined the mutual role of EGFR and sortilin in their nuclear translocation. EGFR silencing significantly reduced the amount of sortilin in nuclear lysate, even after EGF stimulation (*p* < 0.05, Fig. 2a, c), suggesting that sortilin translocation is dependent on EGFR rather than another member of the EGF receptor family. Conversely, when the *SORT1* gene encoding sortilin was depleted in A549 [18], EGFR nuclear translocation was not impaired; in fact, it increased significantly (#, *p* < *0.05*, Fig. 2b, d, e). However, because EGFR remains largely retained at the plasma membrane in *SORT1-*depleted cells [18] (Fig. 2b), the amount of EGFR in the nucleus was significantly lower than in the empty vector control pLKO (*p* < *0.01*, Fig. 2d). Hence, to further investigate the respective roles of EGFR and sortilin in nuclear translocation, we engineered HEK293T knockout cells for either *EGFR* or *SORT1* depletion by using CRISPR/Cas9-mediated gene silencing (Figs. 2f–k). The HEK293T cell line remains a valuable tool for experiments using CRISPR technology due to its high efficiency of transfection, enabling rapid and reliable genetic modifications for mechanistic studies [24]. Hence, we assessed whether sortilin or EGFR is required for mutual translocation into the nuclear fraction. Edited HEK293T cells, either lacking *EGFR* or *SORT1*, were transiently transfected with plasmids encoding wild-type EGFR (EGFRwt) or a mutant form (EGFR S229A) that impairs EGFR phosphorylation and nuclear importation [16] (Fig. 2h, j, k). We also transiently transfected a C-tail truncated version of sortilin (sortilin^∆c^) lacking its retrograde transport from membranes [23] (Fig. 2i–k). As previously evidenced above, EGF stimulation induced significant nuclear translocation of both EGFRwt and sortilin in control cells (Fig. 2g, j, k). In contrast, in HEK293T cells lacking EGFR (HEK CRISPR^*EGFR*^), sortilin remained in the post-nuclear supernatant despite EGF stimulation (Fig. 2f). Likewise, in cells expressing the EGFR mutant S229A, known to inhibit EGFR nuclear translocation [16], sortilin failed to be translocated into the nucleus (Fig. 2h, J). Therefore, we expressed the sortilin^Δc^ form in cells with a sortilin-null background (HEK CRISPR^*SORT1*^) and then stimulated them with EGF. We observed that the nuclear translocation of EGFR wt was unaltered, suggesting that EGFR can translocate to the nucleus independently of sortilin.

Together, these findings indicate that sortilin did not mediate EGFR trafficking to the nucleus in response to EGF stimulation but likely plays a key role once it complexes with EGFR in the nucleus. Importantly, EGFR nuclear import seems to be required for sortilin translocation. Hence, aggregation of EGFR–sortilin complexes in the nuclei of EGF-stimulated cells suggests a specific sub-nuclear localization that might be involved in transcriptional functions [25].

### EGFR–sortilin complexes co-immunoprecipitate with chromatin

**Fig. 3:**
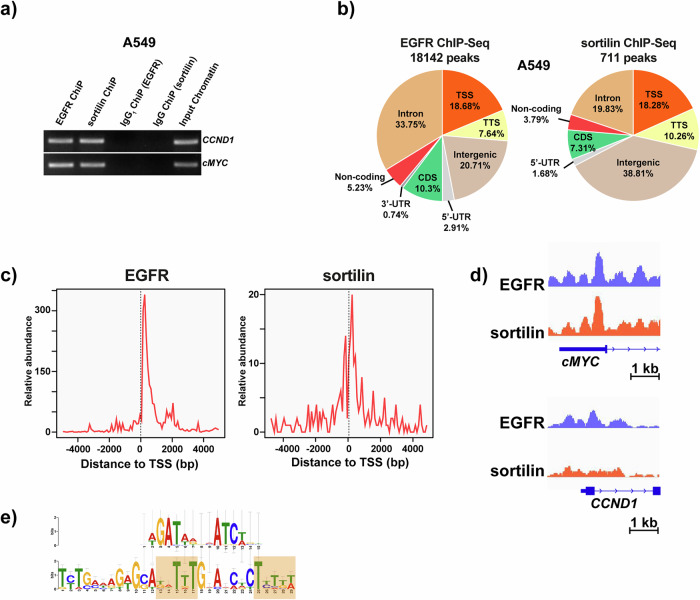
EGFR and sortilin interact with chromatin. **a** PCR amplification of *CCND1* and *cMYC* promoter sequences in A549 cells stimulated with EGF (50 ng/mL) for 30 min following chromatin immunoprecipitation (ChIP) with either anti-EGFR or anti-sortilin antibodies. Respective isotype IgGs, IgG1 ChIP (EGFR) and IgG ChIP (sortilin), were used as controls and compared with input samples (input chromatin), corresponding to non-ChIP DNA, as an internal control. **b** Peaks enriched for EGFR and sortilin in A549 cells stimulated with EGF (50 ng/mL) for 30 min. TTS Transcription Termination Site, TSS Transcription Starting Site, CDS Coding Sequence, UTR Untranslated Region (**c**) Distribution of EGFR and sortilin ChIP-Seq reads near 5 kb upstream/downstream of TSS. **d** ChiP-Seq overview with IGV genome browser showing peaks of DNA-binding pattern of EGFR and sortilin on *CCND1* and *cMYC* TSS. **e** Nucleotides motifs enriched in DNA-binding sequences from EGFR or sortilin immunoprecipitated peaks.

To understand the role of nuclear EGFR–sortilin complexes in EGF-stimulated cells, we investigated their chromatin binding properties using ChIP assays combined with micrococcal nucleases (Mnase) digestion and release of mono-nucleosome (Supplementary Material 1a–d). Likewise, the specificity of anti-EGFR and anti-sortilin antibodies was assessed in HEK cells with knockout of either *EGFR* or *SORT1* (supplementary fig. 1). Although sortilin has not been previously shown to act as a transcriptional co-factor, we hypothesized that its nuclear import could be mediated by its interaction with EGFR (Fig. 1i, j). Since the transcriptional pattern of nuclear EGFR is already known, we analyzed specific DNA sequences located within the EGFR promoter regions to determine whether sortilin might interact with and regulate these regions [26]. We selected two EGFR-targeted genes to evaluate the impact of sortilin interaction with EGFR promoter: *cMYC*, a key gene involved in epigenetic reprogramming [27], and *CCDN1*, a critical regulator of cell cycle progression [15]. ChIP assays using anti-EGFR or anti-sortilin antibodies showed that EGF stimulation resulted in the amplification of *cMYC* and *CCND1* chromatin sequences (Fig. 3a). No amplification was observed following immunoprecipitation with the respective isotype controls (IgG1 ChIP EGFR and IgG ChIP sortilin), suggesting that both EGFR and sortilin interact specifically with chromatin and could participate in the regulation of EGF-induced genes (Fig. 3a).

To identify the DNA regions immunoprecipitated by anti-EGFR and anti-sortilin antibodies, the ChIP products were sequenced (ChIP-Seq). All libraries bound by these antibodies met all ChIP-Seq quality control criteria (Supplementary Materials 1b, c). ChIP-Seq experiments were performed on biological replicates following stimulation with 50 ng/mL EGF for 30 min, with reads averaging 50 million. In stimulated A549 cells, the peaks are predominantly enriched and distributed within intergenic and intronic regions, as well as toward transcriptional regulating elements, including the TSS and the transcription termination site (TTS) (Fig. 3b). Analysis of the segmentation of TSS sequences revealed a preferential distribution for EGFR and sortilin. ChIP-Seq peak distributions within 5 kb of TSS with aggregation plots showed that the TSS/TTS ratios for EGFR and sortilin were 2.44 and 1.78, respectively (Supplementary Fig. 2). Not surprisingly, we observed an abundance of TSS peaks co-occurring with the highest expression of EGFR (Fig. 3c and Supplementary Fig. 2). Likewise, their overlap positions in close proximity to the TSS region suggested that EGFR–sortilin complexes affected gene activity (Supplementary Fig. 2). The PLA and co-immunoprecipitation assays showing the physical interactions between EGFR and sortilin in the nuclei of A549 cells (Fig. 1a–c, i, j) suggested that EGFR and sortilin have common binding sites on target loci. Significant correlations between EGFR and sortilin profiles were shown on ChiP-Seq overview using the Integrative Genomics Viewer (IGV) genome browser [28], which found EGFR and sortilin binding sites on the *CCND1* and *cMYC* TSS (Fig. 3d). Similarly, in silico analysis suggested that both EGFR and sortilin bound to an AT-rich minimal consensus sequence (ATRS), consisting of TNTTT or TTTNT, with N being any nucleotide (Fig. 3e). These genomic sequences were previously associated with the EGFR chromatin binding site [15, 26], suggesting that EGFR–sortilin complexes potentially bind chromatin through EGFR. However, these results cannot exclude that sortilin may bind chromatin itself in the region of the ATRS sequences.

Taken together with our previous results, the binding patterns of EGFR and sortilin were close to gene-proximal regulatory elements, particularly those associated with EGF-induced molecular processes. The proximity of these complexes to key transcriptional regions, such as the TSS, and their potential to bind to ATRS sequences, indicate that EGFR–sortilin complexes likely play an important role in regulating the transcription of EGF-responsive genes, such as *cMYC* and *CCND1*.

### EGF stimulation enhances sortilin DNA binding

**Fig. 4:**
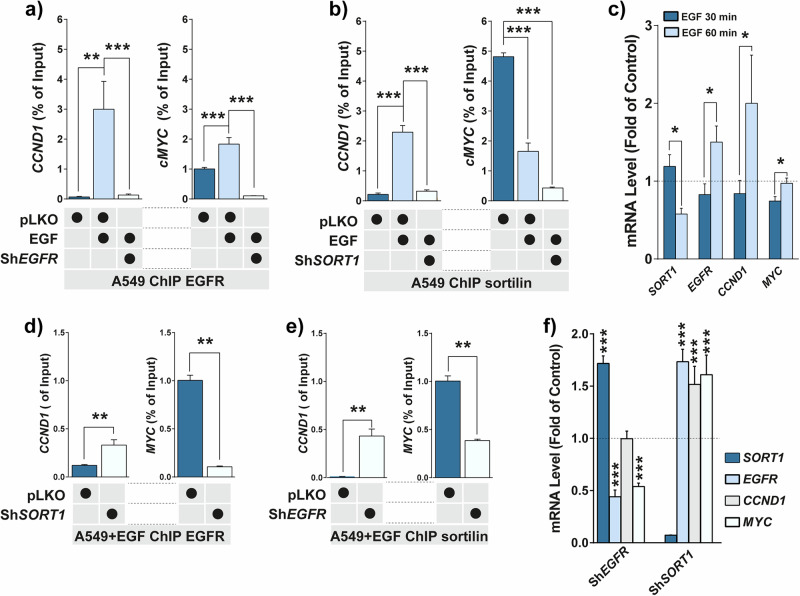
EGF stimulation increases EGFR and sortilin binding to chromatin. **a**, **b** Quantitative PCR (qPCR) of chromatin immunoprecipitated (ChIP) by anti-EGFR or anti-sortilin antibodies in empty vector control cells (pLKO) and cells depleted respectively for *EGFR* or *SORT1* mRNA (shRNAs), and incubated in the absence or presence of EGF (50 ng/mL) for 30 min. Histograms represented the percentages of input following normalization. *CCND1* and *cMYC* promoters were amplified by qPCR. **c** mRNA level (fold of control: EGF non-stimulated cells) of *SORT1*, *EGFR*, *CCDN1* and *MYC* in A549 cells following 30 (dark blue histogram) and 60 min (light blue histogram) of EGF stimulation (50 ng/mL). **d**, **e** Results of EGFR and sortilin ChIP-qPCR of A549 cells depleted or not, either for *SORT1* (Sh*SORT1*) or *EGFR* (*ShEGFR),* incubated in the presence of EGF (50 ng/mL for 30 min). CCND1 and cMYC promoter sequences were amplified by qPCR. **f** mRNA level (fold of control: EGF non-stimulated cells) of *SORT1* (dark blue histogram), *EGFR* (light blue histogram), *CCDN1* (gray histogram) and c*MYC* (white histogram) in A549 cells following 30 min of EGF stimulation (50 ng/mL).

To further assess whether EGF promotes EGFR and sortilin DNA binding to transcriptional regulatory elements, we designed primers corresponding to the TSS regions of genes derived from gene ontology (GO) analysis (Supplementary Table 4), followed by the use of immunoprecipitated chromatin as a qPCR template. A549 cells were respectively depleted for *EGFR* (Fig. 4a) and *SORT1* (Fig. 4b) mRNAs [18, 19] using specific lentivirus delivering shRNAs. Then, depleted cells were incubated with antibodies to specifically immunoprecipitate chromatin. EGF stimulation triggered significant (*p* < *0.001*) chromatin binding of EGFR onto the TSS regions derived from *CCND1*, *cMYC*, and several genes selected by GO analysis (Fig. 4a and Supplementary Fig. 3a). EGF stimulation significantly enhanced sortilin binding to the TSS of selected genes (Fig. 4b and Supplementary Fig. 3b). On the contrary, its binding to *cMYC* TSS was decreased following EGF stimulation compared with control cells (Fig. 4b). Thus, we assessed mRNA level of *SORT1*, *EGFR*, *CCND1* and *cMYC*, 30 or 60 min of EGF stimulation (Fig. 4c). Following 60 min of EGF stimulation both *EGFR* and *CCND1* mRNA level increased significantly (*p* < *0.05*) whereas *cMYC* mRNA level increased more slowly to reach its basal level. Conversely, *SORT1* mRNA levels remain sensitive to EGFR transcriptional program following EGF stimulation [29]. Since both *SORT1* mRNA level and its binding to *cMYC* TSS decreased, these results would suggest that sortilin is involved in *cMYC* gene inhibition. Hence, we assessed whether sortilin or EGFR binds the chromatin. Interestingly, by using either sortilin or EGFR-depleted cell lines, EGF stimulation triggers an increase of EGFR and sortilin binding to *CCND1* TSS, respectively, in *SORT1* and *EGFR*-depleted cell lines (Fig. 4d, e). In addition, their binding to *cMYC* TSS decreased, suggesting that EGFR–sortilin complexes would be required especially for *cMYC* gene activity. However, EGFR depletion notably decreased *cMYC* mRNA levels (*p* < *0.001*), whereas *SORT1* depletion led to a significant increase in the mRNA levels of both *EGFR*, *cMYC* and *CCND1* (*p* < 0.001). These results suggest that sortilin plays a role in the transcriptional regulation of these genes, likely by limiting EGFR transcriptional activity (Fig. 4f). These results suggest that sortilin binding in normal cells by competing with *EGFR* potentially limits *cMYC* and *CCDN1* gene activity. Consequently, these findings indicate that changes in *cMYC* expression could depend on EGFR (activating) and sortilin (inhibiting) levels, confirming their antagonistic and competitive roles in regulating *cMYC* mRNA expression.

Taken together, these findings suggest that sortilin acts as an inhibitor of EGFR-driven gene activation, particularly for *cMYC*, by limiting the transcriptional activity of EGFR and acting as a nuclear antagonist. In this capacity, sortilin likely plays a tumor suppressor-like role in LUAD by modulating the transcriptional network activated by EGFR, thereby controlling the expression of key oncogenic drivers.

### Sortilin overexpression limits polymerase II recruitment to TSS

**Fig. 5:**
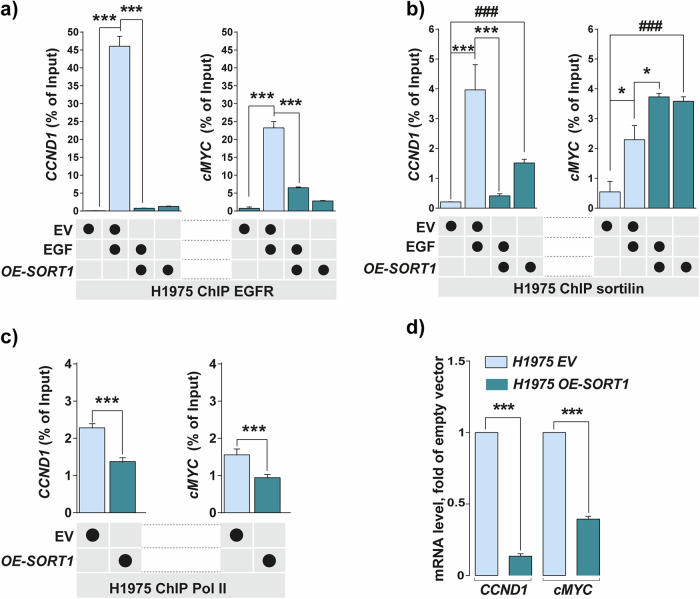
Sortilin overexpression increases sortilin chromatin binding on *cMYC* and *CCND1* and limits polymerase II recruitment. **a**, **b** EGFR and sortilin ChIP-qPCR were performed on H1975 control cells transfected with control empty vector (EV) or on H1975 sortilin overexpressing cells (*OE-SORT1*) in the absence or presence of EGF (50 ng/mL) for 30 min. *CCND1* and *cMYC* promoters were amplified by qPCR. **c** Pol II ChIP-qPCR performed on EV and *OE-SORT1* cells. **d** Levels of *CCND1* and *cMYC* mRNAs in control empty vector (EV) and *OE-SORT1* cells by qPCR. All values represent means ± SD. *p < 0.05, **p < 0.01, ***p < 0.001, ^#^*p* < 0.05, ^##^*p* < 0.01 and ^###^*p* < 0.001 by Student’s *t* test^.^ Each experiment was repeated at least three times.

Hence, to evaluate whether changes in sortilin expression could be involved in EGFR transcriptional activity, we performed ChIP experiments using H1975 cells. This cell line harbors the EGFR T790M mutation, particularly suitable for studying the effects of EGFR inhibitors like osimertinib. However, this cell line harbors the weaker expression of sortilin and thereby offers the possibility to overexpress *SORT1* (*OE-SORT1*) and study the specific impact of EGFR T790M mutation on EGFR and sortilin dynamics in comparison to control cells with control empty vector (EV). As expected, EGF stimulation of control (EV) cells triggered significant (*p* < *0.001*) chromatin binding by both EGFR and endogenous sortilin (Fig. 5a, b and Supplementary Fig. 3a, b). By contrast, sortilin overexpression significantly reduced EGFR DNA binding on *CCND1* and *cMYC* despite EGF stimulation (Fig. 5a and Supplementary Fig. 4a). Under such experimental conditions in H1975 cells, EGFR chromatin binding to *CCND1* and *cMYC* TSS was reduced in comparison to EGF-stimulated cells. By contrast, sortilin binding to the *cMYC* TSS is slightly increased by EGF stimulation (Fig. 5b). Sortilin binding to the *cMYC* and *CCND1* TSS was increased in *SORT1*-overexpressing cells compared to empty vector control (EV) cells. Thus, sortilin continued to occupy the *cMYC* TSS when compared with non-stimulated *OE-SORT1* cells (Fig. 5b and Supplementary Fig. 4b). Using this model, we assessed the recruitment of polymerase II (Pol II), belonging to the initiating transcription complex. Interestingly, the chromatin binding of Pol II to *cMYC* and *CCND1* TSS was significantly lower in cells overexpressing sortilin than in control cells, as was the binding of Pol II to selected genes from GO analysis (Fig. 5c and Supplementary Fig. 4c). These results suggest that sortilin might impair the recruitment of Pol II, thereby influencing *CCND1* and *cMYC* expression. Hence, to further evaluate the consequences of increased sortilin chromatin binding, we measured the levels in these cells of *CCND1* and *cMYC* mRNAs. Surprisingly, sortilin overexpression significantly reduced (*p* < *0.001*) the mRNA levels of the EGFR co-drivers *CCND1* and *cMYC* (Fig. 5d).

Taken together, these results suggest that the amount of sortilin would represent a limiting factor to impair EGFR chromatin binding and Pol II recruitment at the TSS of EGF response genes.

### Osimertinib triggers nuclear importation of EGFR

Given the link between EGFR nuclear translocation and TKI resistance [9, 16, 30], we studied sortilin function in TKI-treated H1975 cells with EGFR T790M mutations and extended our analysis to H3255 cells harboring an L858R activating mutation. EGFR’s spatial and temporal distribution impacts relapse risks due to sustained signaling or enhanced nuclear importation. Notably, EGFR subcellular distribution varies depending on its mutational status; for instance, in H1975 cells harboring both the L858R and T790M mutations, EGFR is constitutively active and undergoes internalization even in the absence of ligand stimulation [18, 31]. We investigated whether osimertinib exposure, a TKI targeting the EGFR T790M mutation or the L858R mutation indirectly, affects EGFR chromatin binding in these two models. Likewise, we assess whether the anti-cancer properties of this TKI are limited by the competition between EGFR and sortilin for chromatin binding.

**Fig. 6:**
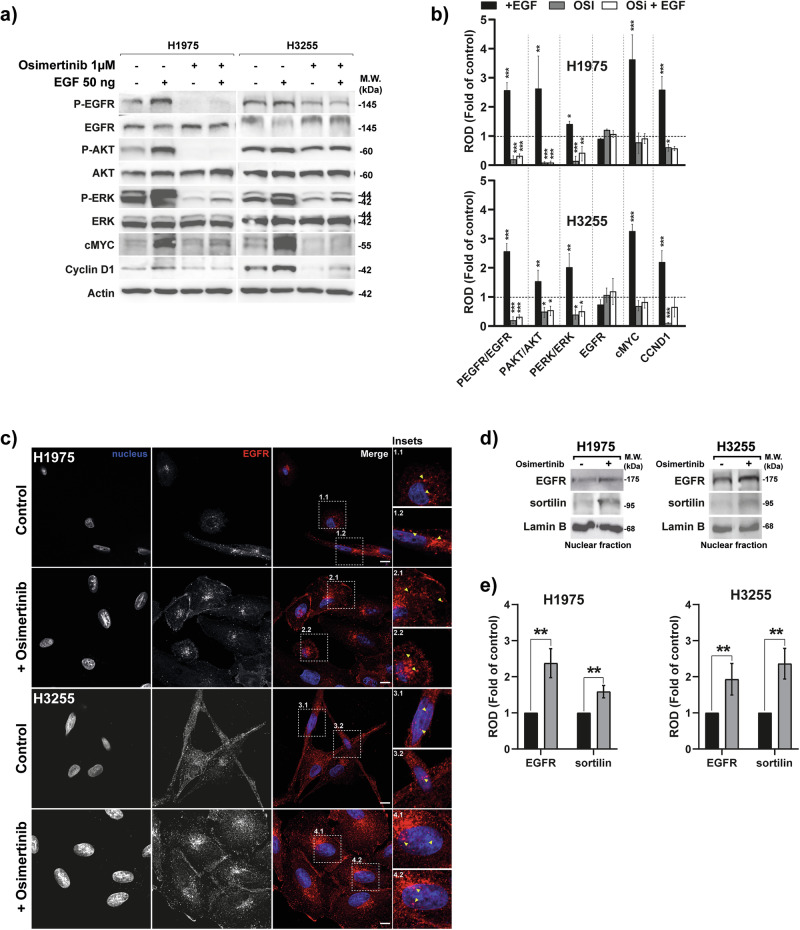
Osimertinib enhances nuclear importation of EGFR. **a** Western blot analysis of phospho-EGFR, total EGFR, phospho-AKT, total AKT, phospho-ERK, total ERK, sortilin, MYC and cyclin D1 in H1975 and H3255 cells in the absence or presence of EGF (50 ng/mL) for 30 min, treated or not with osimertinib (1 μM) for 24 h. Actin serves as a loading control. **b** Densitometric quantification in relative optical density (ROD) of p-EGFR/total EGFR, p-AKT/total AKT, and p-ERK/total ERK ratios, normalized to untreated controls (fold of control, indicated by dotted line). All values represent means ± SD. *p < 0.05, **p < 0.01, ***p < 0.001, by Student’s *t* test. Each experiment was repeated at least three times. **c** EGFR localization was analyzed by confocal microscopy in H1975 and H3255 cells, in the absence or presence of osimertinib treatment (1 µM for 24 h). Scale bar, 10 μm yellow arrows show EGFR localization. **d** Western blotting performed on nuclear fraction showing that treatment of H1975 and H3255 cells with osimertinib (1 µM for 24 h) triggers EGFR and sortilin importation into cell nuclei. Lamin B was used as a loading control for the nuclear fraction. Molecular weight (MW) in kilodaltons (kDa). **e** Densitometric quantification in relative optical density (ROD) of total EGFR, and sortilin, normalized to untreated controls (fold of control). All values represent means ± SD. *p < 0.05, **p < 0.01, ***p < 0.001, by Student’s *t* test. Each experiment was repeated at least three times.

As attempted, 24 h of osimertinib treatment triggers a significant decrease in EGFR phosphorylation at concentrations as low as 0.01 µM for both cell lines (Fig. 6a, b) and suppresses AKT and ERK phosphorylation (fold of control) in the presence or absence of EGF stimulation in both cell lines (Fig. 6a, b). Notably, a complete inhibition of EGFR phosphorylation was observed at 1 µM osimertinib in the H1975 cell line, which was the effect we sought to achieve in this model. Consistent with these findings, a clear dose-dependent inhibition of EGFR phosphorylation was illustrated (Supplementary Fig. 5a–d) and a marked reduction in cell viability, particularly upon sortilin overexpression induced by doxycycline in both cell lines (Supplementary method 1 and Supplementary Fig. 5e, g). To mimic physiological conditions, we used EGF stimulation since EGF binding can promote receptor internalization [32]. Our results also confirm that EGF stimulation induces a decrease of L858R and L858R/T790M mutated EGFR in H3255 and H1975 cell lines, as already described [33] (Fig. 6a, b). Additionally, osimertinib treatment led to a marked reduction in cyclin D1 expression, further supporting the suppression of key signaling pathways associated with cell proliferation. Strikingly, while the T790M mutant EGFR remains internalized under control conditions (Fig. 6c, insets 1.1, 1.2 and 3.1, 3.2), we found that treating H1975 and H3255 cells with 1 µM osimertinib for 24 h triggered EGFR subcellular relocation toward the nuclei of exposed cells (Fig. 6c, insets 2.1, 2.2 and 4.1, 4.2). Cell fractionation and isolation of nuclei confirmed a significant importation of EGFR into the nuclei (Fig. 6d, e), despite a reduction in its kinase activity, as evidenced by decreased phosphorylation levels (P-EGFR) (Fig. 6a, b). Importantly, under these conditions of complete EGFR phosphorylation inhibition, the phenotypic and transcriptional effects we observed can be attributed specifically to EGFR inhibition rather than off-target actions (Fig. 6a, b and Supplementary Fig. 5a–d).

**Fig. 7:**
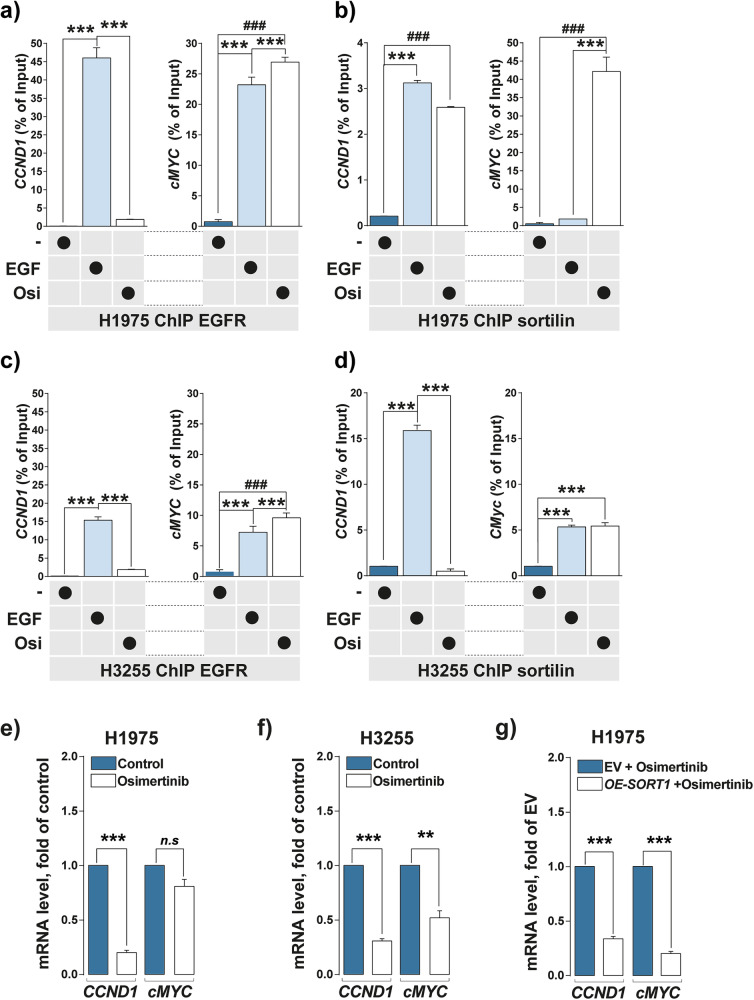
Osimertinib modulates EGFR and sortilin chromatin binding. **a**–**d** Quantitative PCR (qPCR) of chromatin immunoprecipitated (ChIP) by anti-EGFR or anti-sortilin antibodies in H1975 and H3255 cells incubated in the absence or presence of EGF (50 ng/mL) for 30 min or osimertinib (1 µM) for 24 h. Histograms represented the percentages of input following normalization. *CCND1* and *cMYC* promoters were amplified by qPCR. **e**, **f** mRNA level (fold of control: osimertinib non-treated cells) of *CCDN1* and *MYC* in H1975 and H3255 cells following osimertinib treatment (1 µM) for 24 h. **g** mRNA level (fold of control: osimertinib non-treated cells) of *CCDN1* and *MYC* in control H1975 cells carrying empty vector (EV) and H1975 cells overexpressing (*OE-SORT1*) in the presence of osimertinib (1 µM for 24 h). All values represent means ± SD. *p < 0.05, **p < 0.01, ***p < 0.001, ^#^*p* < 0.05, ^##^*p* < 0.01 and ^###^*p* < 0.001 by Student’s *t* test^.^ Each experiment was repeated at least three times.

Then, we subsequently assessed whether EGFR binding to chromatin increases following its enrichment in the nuclear compartment. ChIP EGFR in H1975 and H3255 cells showed that only *cMYC* TSS binding was significantly increased following osimertinib treatment in both cell lines (Fig. 7a, c and Supplementary Fig. 6a). Strikingly, under the same conditions, ChIP analysis of sortilin revealed that osimertinib treatment significantly increased the binding of sortilin to *CCND1*, *cMYC*, and selected genes identified from GO analysis in H1975 whereas only *cMYC* TSS binding was significantly increased in H3255 cells. (Fig. 7b, d and Supplementary Fig. 6b). To further analyze gene activity following chromatin binding by EGFR and sortilin, we assessed the levels of *CCND1* and *cMYC* mRNAs in these cells (Fig. 7e, f). Osimertinib treatment of H1975 cells did not significantly reduce the level of *cMYC* mRNA relative to that of *CCND1* mRNA (Fig. 7e), whereas both transcripts decreased in the H3255 cells (Fig. 7f). However, when H1975 cells overexpress *SORT1 (OE*-*SORT1*), we observed that osimertinib induced a significant decrease in *cMYC* mRNA level in comparison to control EV cells (Fig. 7g). These results would suggest that the mutational status of NSCLC cell lines might influence the regulation of *cMYC*. Furthermore, while no significant variation was observed in cMYC protein expression, Cyclin D1 protein levels were strongly decreased in the presence of osimertinib alone in both H1975 and H3255 cell lines. This decrease in Cyclin D1 occurred concurrently with the inactivation of EGFR, AKT and ERK phosphorylation (Fig. 6a, b).

Taken together, these results indicate that EGFR and sortilin compete for binding to the regulatory elements of the *cMYC* gene, and that the nuclear expression of sortilin may be a limiting factor in counteracting the transcriptional program driven by *EGFR*.

### Inverse correlation between cMYC and sortilin expression

**Fig. 8:**
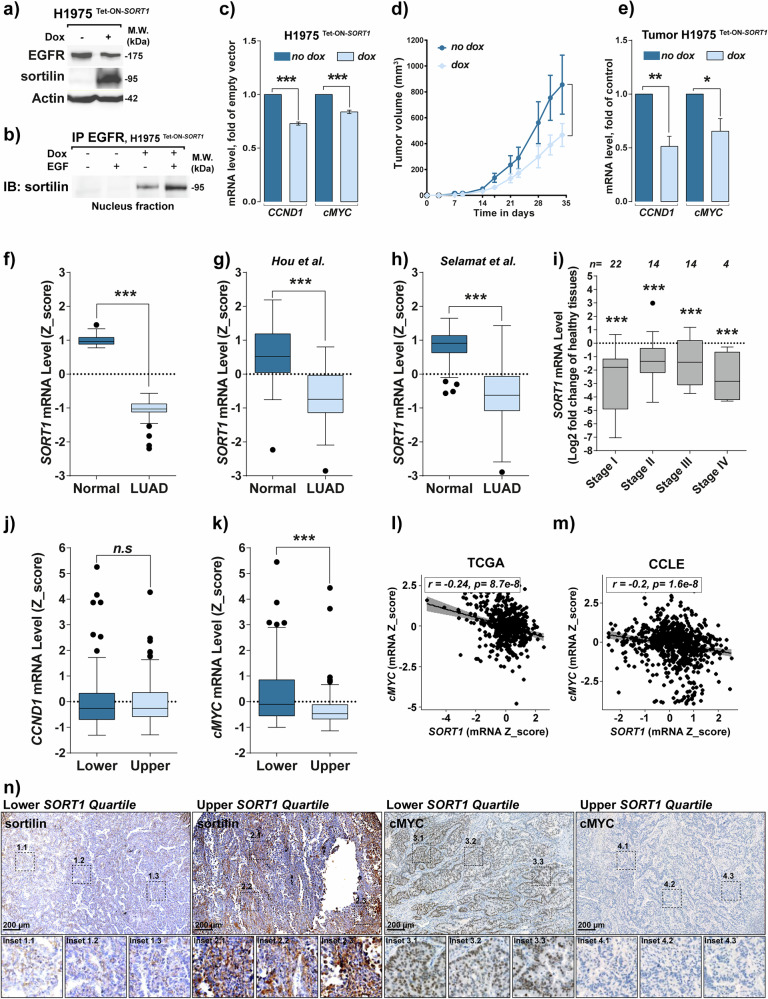
*cMYC* expression correlates inversely with *SORT1* expression in vitro and in tumor samples. **a** Western blotting showing EGFR and sortilin expression in lysates of H1975^Tet-ON-*SORT1*^ cells following incubation in the absence or presence of 100 nM doxycyclin (dox) for 24 h. **b** Anti-EGFR immunoprecipitation (IP) of isolated nuclei from H1975^Tet-ON-*SORT1*^ cells following incubation in the absence or presence of 100 nM doxycyclin for 24 h and stimulation with 50 ng/mL EGF for 30 min and immunoblotting (IB) with anti-sortilin. **c** Comparison of *CCND1* and *cMYC* mRNA levels in H1975^Tet-ON-*SORT1*^ cells following incubation in the absence or presence of 100 nM doxycycline for 24 h. **d** Effects of doxycycline on tumor induction by H1975^Tet-ON-*SORT1*^ cells in NOD-SCID mice. H1975^Tet-ON-*SORT1*^ cells were subcutaneously engrafted (3 × 10^6^ cells/mouse) onto NOD-SCID mice. Fifteen days later, corresponding to the beginning of tumor development, mice were treated with 2 mg/mL doxycycline in drinking water or drinking water alone, and tumor volumes were measured. Tumor growth curves are shown for mice treated with dox (light blue curve) and for control mice (blue curve). **e** qPCR measurements of expression of *CCND1* and *cMYC* mRNAs in tumors of mice treated with (light blue bar) or without (dark blue bar) dox. Measurements of *SORT1* mRNA levels (Z-score) in normal and lung adenocarcinoma (ADC) tissue samples obtained from the (**f**) Limoges University Hospital cohort and data sets from **g** Hou et al. and **h** Salamat et al. (**i**) qPCR measurements of *SORT1* mRNA levels in tumor samples compared to healthy tissues from the Limoges University Hospital cohort at different stages. Quantification of (**j**) *CCND1* and **k** *cMYC* mRNA levels in tumor samples from the Limoges University Hospital cohort expressing the lowest and highest quartiles of sortilin expression. **l** Correlation between levels of *cMYC* and *SORT1* mRNA levels in NSCLC patients in the TCGA database (*r* = –0.24; *p* = 8.7.10^–8^) and **m** in solid cancer cell lines from the Cancer Cell Line Encyclopedia (CCLE) database (*r* = -0.2; *p* = 1.6.10^–8^). Diagrams represent the correlation between *SORT1* expression and *cMYC* expression. All values are expressed as means ± SD, **p < 0.01 and ***p < 0.001 by Student’s *t* test, n.s. not significant. Each experiment was repeated at least three times. **n** Representative immunohistochemical staining of sortilin and cMYC in lung tumor samples from the lower and upper quartiles of *SORT1* mRNA expression. Scale bars represent 200 μm.

Because uncontrolled EGFR signaling leads to cell transformation [3, 34] and LUAD initiation [35–37], we developed a Tet-ON inducible model to trigger sortilin expression in H1975 cells. Doxycycline (Dox) treatment increased sortilin expression and slightly decreased EGFR (Fig. 8a). EGFR immunoprecipitation showed increased EGFR–sortilin complexes in nuclei of induced cells, with or without EGF stimulation (Fig. 8b). Interestingly, levels of *CCND1* and *cMYC* mRNAs decreased significantly following Dox stimulation (*p* < *0.001*, Fig. 8c). In vivo, Dox-treated mice with induced tumors showed significantly slower tumor progression (*p* < 0.001, Fig. 8d) and reduced *CCND1* (*p* < 0.01) and *cMYC* (*p* < 0.05) mRNAs (Fig. 8e). These results suggest that sortilin may repress EGFR-regulated genes upon EGF stimulation. Analyzing *SORT1* mRNA expression in 54 LUAD tumors, obtained from the Tumor Bank (CRBiolim) of Limoges University Hospital, under protocols approved by the IRB (AC-2013-1853, DC-2011-1264), we found significantly lower levels in tumors compared to normal tissue (*p* < *0.001*, Fig. 8f). This finding was confirmed in two other studies [38, 39] (*p* < 0.001, Fig. 8g, h), regardless of disease stage (Fig. 8i). By sorting patients by *SORT1* mRNA quartiles, we observed that only *cMYC* mRNA expression significantly decreased in the highest quartiles of *SORT1* mRNA (*p* < *0.001*, Fig. 8j, k). We evaluated sortilin effect on *cMYC* expression in MSKCC cBioPortal data sets [40, 41], including 240 TCGA patients (Fig. 8l) [42] and 665 CCLE cancer cell lines (Fig. 8m) [43]. *cMYC* expression was inversely correlated with *SORT1* in both patient tissues (*r* = –0.24, *p* = 8.7e-8) and cell lines (*r* = –0.2, *p* = 1.6e-8) while no such correlation between *SORT1* mRNA and *CCND1* mRNA was observed.

Immunohistochemical analysis of cMYC expression was performed on lung tumor samples stratified into the lower and upper quartiles of sortilin expression. We observed a significant inverse correlation between sortilin and cMYC expression levels. Tumors with low sortilin expression exhibited high cMYC immunoreactivity (Fig. 8n, insets 1.1 to 1.3, and 3.1 to 3.3), whereas tumors with high sortilin expression related to the upper quartile showed low cMYC staining intensity (Fig. 8n, insets 2.1 to 2.3 and 4.1 to 4.2).

These findings suggest that sortilin impairs EGFR-regulated *cMYC* transcription. Likewise, in lung malignant tissues, sortilin is often downregulated, which disrupts its normal tumor-suppressive function of limiting EGFR transcriptional responses. This loss of sortilin-mediated regulation potentially enhances tumor malignancy, particularly in the context of mutant EGFR, and may contribute to reduced efficacy of TKI treatment.

## Discussion

The present study demonstrated that sortilin is a key regulator of nuclear EGFR, limiting its transducing activity. Sortilin interacts with EGFR at the chromatin regulatory elements of EGF response genes, such as those involved in cell reprogramming (*cMYC*) and proliferation (*CCND1*), both of which are hallmarks of cancer [44]. These findings highlight sortilin function at the nuclear level in downregulating EGFR oncogenic co-drivers. The study revealed that sortilin not only directs EGFR toward rapid internalization and degradation following EGF stimulation [18] but also suppresses its function directly at the nuclear level. Indeed, our results emphasize that sortilin is an EGFR antagonist, by not only limiting EGFR signaling at the plasma membrane but also modulating its transcriptional activity in the nucleus. Among them, previous reports have described that nuclear EGFR upregulates *CCND1* [26] gene expression notably in patients' lung tumors harboring EGFR mutation [45]. Therefore, our results demonstrating that *CCDN1* and *cMYC* are downregulated when sortilin is overexpressed strengthen that sortilin directly or indirectly acts as an EGFR antagonist. Using PLA and nuclear IP immunoprecipitation experiments, the spatiotemporal distribution of EGFR and sortilin in the nuclei of EGF-stimulated cells was detected. Chromatin immunoprecipitation and genome-wide analysis showed that EGFR and sortilin bind to the same regulatory elements of EGF response genes [46, 47], particularly at TSS containing the EGFR binding chromatin sequence ATRS [15, 26]. Strikingly, EGFR-sortilin interaction was recently described in head and neck cancer, where it appears to interfere with the pro-oncogenic signaling of EGF and improve overall survival. Interestingly, sortilin alone decreases overall survival, whereas the complex EGFR/sortilin increases it and is significantly associated with better prognosis as demonstrated with PLA-EGFR/ sortilin in tumor microarray [48]. Our results support this hypothesis and suggest a novel mechanism in which the interaction between EGFR and sortilin may be necessary for promoting the nuclear import of sortilin and trigger its interaction with EGFR binding chromatin sequence. Given that sortilin expression in LUAD cell lines is low, the role of sortilin in the EGFR transcriptional program was studied using both constitutive and inducible models of *SORT1* expression in the highly aggressive H1975 cell line with the T790M EGFR mutation. Although sortilin promotes EGFR internalization [18, 19], *SORT1* overexpression was found to enhance its binding to chromatin. This resulted in decreased binding of EGFR and Pol II to the TSS of *cMYC* and *CCND1*, leading to reduced mRNA levels of these genes. In vivo, *SORT1* expression slowed tumor progression and significantly reduced *cMYC* and *CCND1* mRNA levels. These results suggest that sortilin binds TSS sequences irrespective of stimuli, and its nuclear expression is crucial for limiting EGFR nuclear networking. To better understand the differential regulation of CCND1 and cMYC observed in our study, we propose several hypotheses: First, *CCND1* transcription may be more directly dependent on EGFR kinase activity than *cMYC*, suggesting a differential sensitivity to EGFR inhibition in H1975 cells. Second, compensatory mechanisms involving other transcription factors or signaling pathways might sustain *cMYC* expression even in the presence of EGFR inhibition. Finally, sortilin itself may play a more prominent role in repressing *CCND1* transcription compared to *cMYC*, possibly due to variations in promoter architecture or the recruitment of specific cofactors. These hypotheses provide a framework for understanding the nuanced interplay between EGFR, sortilin, and downstream targets like *CCND1* and *cMYC*. These findings also indicate that expression of sortilin is a limiting factor that restricts EGFR transcriptional activity. These findings suggest that the interaction between nuclear EGFR and sortilin could serve as a critical determinant in the cellular response to EGFR-TKIs, with sortilin potentially modulating EGFR transcriptional activity to counteract resistance mechanisms. Likewise, these results would also suggest that sortilin expression would be used as a potential biomarker for predicting response to TKI treatment concerning its limiting function on the proliferative signaling or transcriptional program of EGFR. Indeed, patients who retain either sortilin expression or in complex with EGFR exhibit higher survival rates, particularly among those positive for EGFR mutations [18, 48].

To determine the nuclear localization of sortilin in a model with inactivated EGFR, cells were treated with the TKI osimertinib, which inhibits EGFR kinase activity, limiting its phosphorylation and endocytosis; crucial steps for nuclear importation. In EGFR-mutated LUAD cells, the nuclear import of the EGFR-sortilin complex despite kinase inhibition by osimertinib highlights a potential bypass mechanism, where nuclear EGFR signaling persists and may contribute to adaptive resistance. This molecular mechanism has already been reported in EGFR resistance to TKI and depends on the nuclear translocation of EGFR [30]. Despite the decreased phosphorylation of EGFR following osimertinib treatment, both EGFR and sortilin were imported into the nuclei, increasing their chromatin binding. In H1975 cells with mutated EGFR, osimertinib significantly increased *cMYC* mRNA levels, but *cMYC* expression was notably decreased when sortilin was overexpressed in these cells. Interestingly, it was previously reported that the tumor growth inhibition by osimertinib was related to the decrease of the cMYC levels in NSCLC lines with activating EGFR mutations and correlated with in vivo and ex vivo studies [49]. These data support that osimertinib response is related to the downregulation of cMYC [49]. In addition, our findings indicate that the presence of sortilin in EGFR-mutated cells is required for attenuating the EGFR transcriptional program, particularly in downregulating *cMYC* gene activity, which occurs only with sortilin overexpression. This suggests that sortilin acts as a switch factor for the EGFR transcriptional program in LUAD cell lines. Sortilin expression was found to decrease with tumor stages [18], as suggested by our results demonstrated that sortilin is downregulated in most malignant tissues. Indeed, in a tissue assay of 54 LUAD patients, only *cMYC* mRNA levels significantly decreased in malignant tissues with high *SORT1* mRNA levels. This inverse correlation between *cMYC* and *SORT1* expression was also observed in tumor tissues and solid cancer cell lines in public datasets and in patients’ tumors, as evidenced in this study. Overall, these results highlight sortilin tumor suppressor-like activity, showing that it alters *cMYC* gene activity. *cMYC* co-occurs with oncogenic driver alterations such as EGFR T790M mutation [20]. Since *cMYC* expression reprograms cells, leading to the formation and maintenance of tumor-initiating cells with metastatic capacities, these cells become resistant to both anti-EGFR therapy [11] and radiotherapy [50].

In summary, our findings provide insight into the role of sortilin in LUAD. Sortilin binds to chromatin elements of EGF response genes, thereby repressing *cMYC* transcription. This regulatory mechanism suggests that sortilin expression may predict tumor responses to anti-EGFR treatment and patient outcomes.

However, this study has certain limitations. First, while we demonstrated the regulatory role of sortilin on EGFR transcriptional activity, further functional studies are required to clarify the underlying molecular mechanisms. Second, the use of a limited number of cell models restricts the generalizability of our findings. Future research should validate these results in diverse LUAD models and clinical samples to strengthen their translational potential.

## Supplementary information

Supplementary data

## Data Availability

All relevant data are available from the corresponding authors on request.
