# Supplementary material for: Sortilin exhibits tumor suppressor-like activity by limiting EGFR transduction function
Source: Oncogene. 2026 Feb 14;45(9):875–88. doi: 10.1038/s41388-026-03680-5 (PMC12929063; doi:10.1038/s41388-026-03680-5)
Supplement: Supplementary file 1 — Supplementary data [file 41388_2026_3680_MOESM1_ESM.pdf]

Supplementary Figures

Sortilin exhibits tumor suppressor-like activity by limiting EGFR transducing function

Lapeyronnie E.[*,1], Granet C.[*,1], Tricard J.[*,1,3], Gallet F.[1], Yassine M.[1], Daverat H.[1], Rovini A.[1], Chermat A.[1,3], Jauberteau MO[1], Bertin F.[1,3], Melloni B.[1,2], Vincent F.[1,2], Naves T.[1, §, #] and Lalloué F.[1, §, #]

[1]UMR Inserm 1308 CAPTuR, Contrôle de l'Activation cellulaire, Progression Tumorale et Résistance thérapeutique and Chaire de Pneumologie Expérimentale, Université de Limoges, Faculté de Médecine, 2 Rue du Dr. Raymond Marcland, 87025 Limoges CEDEX-France.

[2]Service de Pathologie Respiratoire, Centre Hospitalier et Universitaire de Limoges, 87042 Limoges CEDEX-France.

[3]Service de Chirurgie Thoracique et Cardio-vasculaire, Centre Hospitalier et Universitaire de Limoges, 87042 Limoges CEDEX-France.

*These authors contributed equally to this work.
[§]Equal contribution.
# corresponding authors

Please address correspondence to:
Thomas Naves
Chaire de Pneumologie Expérimentale
EA3842 CAPTuR, Contrôle de l'Activation cellulaire, Progression Tumorale et Résistance thérapeutique
Faculté de Médecine
2, Rue du Docteur Marcland
87025, Limoges CEDEX
FRANCE
Tel: +33 5 55 45 59 70
Mail: thomas.naves@unilim.fr

Fabrice Lalloué
EA3842 CAPTuR, Contrôle de l'Activation cellulaire, Progression Tumorale et Résistance thérapeutique
Faculté de Médecine
2, Rue du Docteur Marcland
87025, Limoges CEDEX
FRANCE
Tel: +33 5 55 45 59 29
Mail: fabrice.lalloue@unilim.fr

Supplementary Table 1. List of antibodies used

Protein	Reference	Dilution
sortilin	Ab16640, Abcam	1:200
P-EGFR	Tyr-1068, 3777, Cell Signaling	1:1000
EGFR	4267, Cell Signaling	1:1000
EGFR	MA5-13070, Thermofisher	1:500
Histone H3	9715S, Cell Signaling	1:1000
Lamin B1	HPA050524, Atlas Antibodies	1:1000
tubulin	sc-53646, Tebu	1:1000
actin	A2066, Sigma	1:10000
PARP	9532, Cell Signaling	1:1000
Calnexin	2679, Cell Signaling	1:1000
TGN46	T7576, Sigma	1:500
Cyclin D1		
cMYC		

Supplementary Table 2. List of SgRNA used to generate HEK293T cells KO for respectively *SORT1* or *EGFR*

Gene	ensemble ID	Chr	SgRNA Name	SgRNA Sequence	Target	Reverse Sequence
SORT1	ENSG00000134243	1	SgRNA#1	TCATAATTACCAGTGGTACA	Exon 3 (Reverse)	TGTACCACTGGTAATTATGA
SORT1	ENSG00000134243	1	SgRNA#2	ATCTCACCTTCGATATAGCT	Exon 3 (Reverse)	AGCTATATCGAAGGTGAGAT
SORT1	ENSG00000134243	1	SgRNA#3	TTCCCATAATCCTCACTGAG	intron 3-4 and exon 4 (Reverse)	CTCAGTGAGGATTATGGGAA
SORT1	ENSG00000134243	1	SgRNA#4	TCATCAATAACACCTTTATT	Exon 4 (Forward)	
SORT1	ENSG00000134243	1	SgRNA#5	ACTGAATTTGGCATGGCTAT	Exon 4 (Forward)	
EGFR	ENSG00000146648	7	SgRNA#1	GGAGCAGCGATGCGACCCTC	Exon 1 (Forward)	
EGFR	ENSG00000146648	7	SgRNA#2	AGAGCGCAGCCAGCAGCGCC	Exon 1 (Reverse)	GGCGCTGCTGGCTGCGCTCT
EGFR	ENSG00000146648	7	SgRNA#3	CCTCCAGAGCCCGACTCGCC	Exon 1 (Reverse)	GGCGAGTCGGGCTCTGGAGG
EGFR	ENSG00000146648	7	SgRNA#4	AAGGTAAGGGCGTGTCTCGC	Intron 1 (Reverse)	GCGAGACACGCCCTTACCTT
EGFR	ENSG00000146648	7	SgRNA#5	TTACTCGTGCCTTGGCAAAC	Exon 2 (Reverse)	GTTTGCCAAGGCACGAGTAA

Supplementary Table 3. ChIP-qPCR probes were designed to be complementary to the genomic DNA promoter sequence of each targeted gene and were synthesized by the custom TaqMan service from ThermoFisher Scientific

Targeted gene	TaqMan™ probes references
ACTB	Hs01060665_g1
CCND1	Hs00765553_m1
DHODH	Hs00361406_m1
DUSP12	Hs00170898_m1
EGFR	Hs01076090_m1
MYC	Hs00153408_m1
SNAPC1	Hs00608182_m1
SORT1	Hs00361760_m1
STX6	Hs01057343_m1

Supplementary Table 4. Gene ontology (GO) analysis of loci shared by EGFR and sortilin pathway components

GO	Description	Term Type	EGFR *p*-value	Sortilin *p*-value
GO:0007156	Homophilic cell adhesion via plasma membrane adhesion molecule	Biological process	$2.21\ 10^{-11}$	0.03
GO:0098742	Cell–cell adhesion via plasma membrane adhesion molecules	Biological process	$3.67\ 10^{-11}$	0.04
GO:0098609	Cell–cell adhesion	Biological process	$6.70\ 10^{-11}$	0.04
GO:0043169	Cation binding	Molecular function	$2.36\ 10^{-8}$	0.03
GO:0046872	Metal ion binding	Molecular function	$2.87\ 10^{-8}$	0.04
GO:0016020	Membrane	Cellular component	$1\ 10^{-4}$	0.04
GO:0038023	Signaling receptor activity	Molecular function	$4\ 10^{-3}$	0.04
GO:0004888	Transmembrane signaling	Molecular function	$5\ 10^{-3}$	0.02
GO:0016021	Integral component of membrane receptor activity	Cellular component	$9\ 10^{-3}$	0.04
GO:0043565	Sequence-specific DNA binding	Molecular function	0.017	0.01

Supplementary_Materials_1_Lapeyronnie et al.

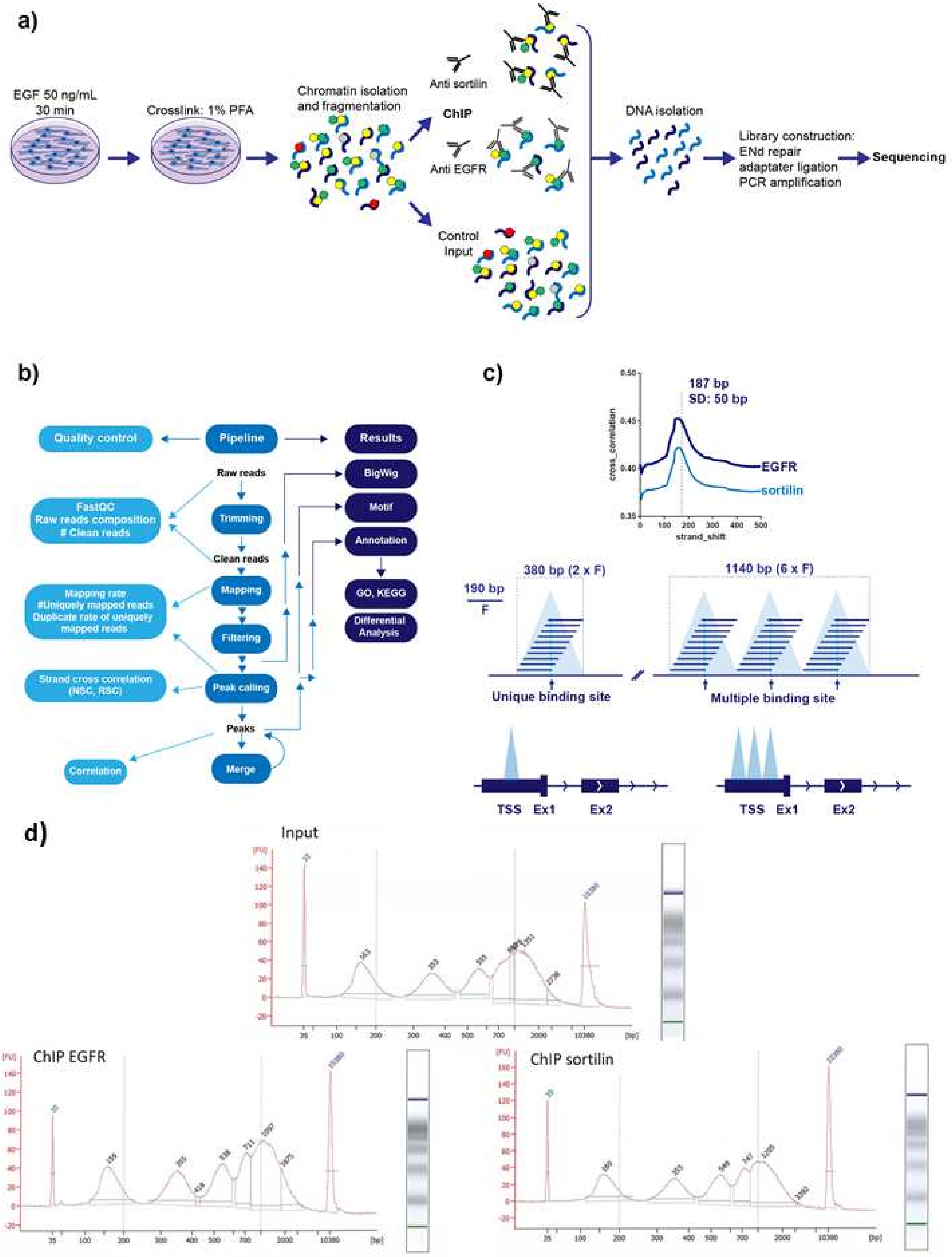

Supplementary material 1: ChIP workflow and bioinformatic pipeline analysis. (a) Schematic showing the ChIP protocol from cells to DNA before sequencing. **(b)** Pipeline of bioinformatic analysis. Chromatin immunoprecipitation sequencing (IP-seq) allows identification of the genome regions that interact efficiently with transcription factors and chromatin-associated proteins. Mapping the sequencing results onto the genome can provide genome-wide information about the DNA regions interacting with histones and transcription factors. **(c)** Determination of unique or multiple DNA binding sites on targeted promoter genes. **(d)** Chromatin digestion by microccocal nuclease after ChIP with an Agilent bioanalyzer to verify the presence of mono-nucleosomes in different sample (Input, ChIP EGFR, and ChIP sortilin).

Supplementary_Method_1_Lapeyronnie et al.

Cell viability assay (XTT)

Cell viability was assessed using the XTT assay (Cell Proliferation Kit II, Roche Applied Science) according to the manufacturer's instructions. Briefly, 3,000 cells per well were seeded in 96-well flat-bottom plates in complete growth medium and allowed to adhere overnight at 37°C in a humidified incubator with 5% CO_2. The following day, cells were treated with osimertinib at the indicated concentrations for 72 h, in the presence or absence of doxycycline (100 nM) when required for sortilin induction. At the end of the treatment period, 50 µL of XTT labeling mixture (prepared according to the manufacturer's protocol) was added to each well containing 100 µL of medium, and the plates were incubated for an additional 2 h at 37°C. Absorbance was measured at 450 nm with a reference wavelength of 650 nm using a microplate reader. Results were normalized to untreated control cells, which were set to 100%. Each condition was tested in triplicate, and data are presented as mean ± SD from at least three independent experiments.

Supplementary_Figure_1_Lapeyronnie et al.

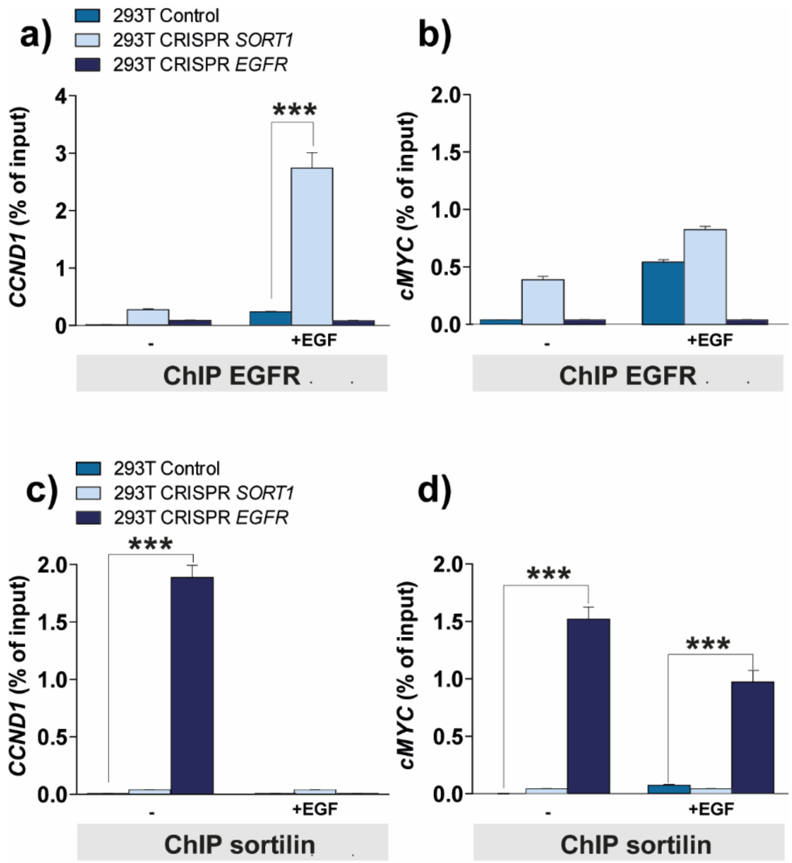

Supplementary Figure 1: Validation of the specificity of anti-EGFR and anti-sortilin antibodies in EGFR or SORT1 CRISPR KO cells by ChIP-qPCR. Quantitative PCR (qPCR) was performed on chromatin immunoprecipitated (ChIP) using anti-EGFR or anti-sortilin antibodies in HEK293T cells edited via CRISPR/Cas9 to knock out *EGFR* or *SORT1*, respectively *293T CRISPR EGFR* (dark blue bar) or *293T CRISPR SORT1* (light blue bar). Cells were incubated in the absence or presence of EGF (50 ng/mL) for 30 minutes. The histograms represent the percentage of input after normalization. *CCND1* and *cMYC* promoters were amplified by qPCR.

Supplementary_Figure_2_Lapeyronnie et al.

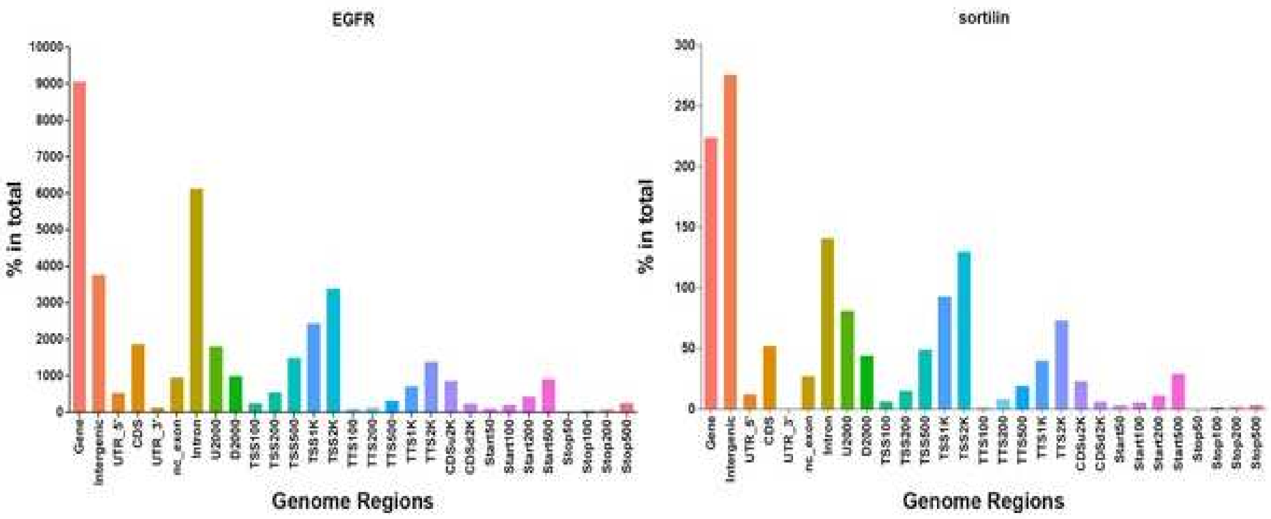

Supplementary Figure 2: Pathways and genome regions targeted by EGFR and sortilin. (a-b) Representations of genome regions targeted by (a) EGFR and (b) sortilin. Distribution of peaks in different functional areas. The horizontal axis represents different functional areas, and the vertical axis represents the ratio of the peak in the functional region to the total peaks. The number at the top of each functional region represents the peak number. U2000 and D2000 indicate 2000 bp in the upstream and downstream regions, respectively; CDSu2K and CDSd2K indicate 2 kb upstream and downstream of the CDS, respectively; and TSS100, TTS100, Start100, and Stop100 indicate the 100 bp regions centered on the TSS, TTS, Start-codon, and Stop-codon, respectively.

Supplementary_Figure_3_Lapeyronnie et al.

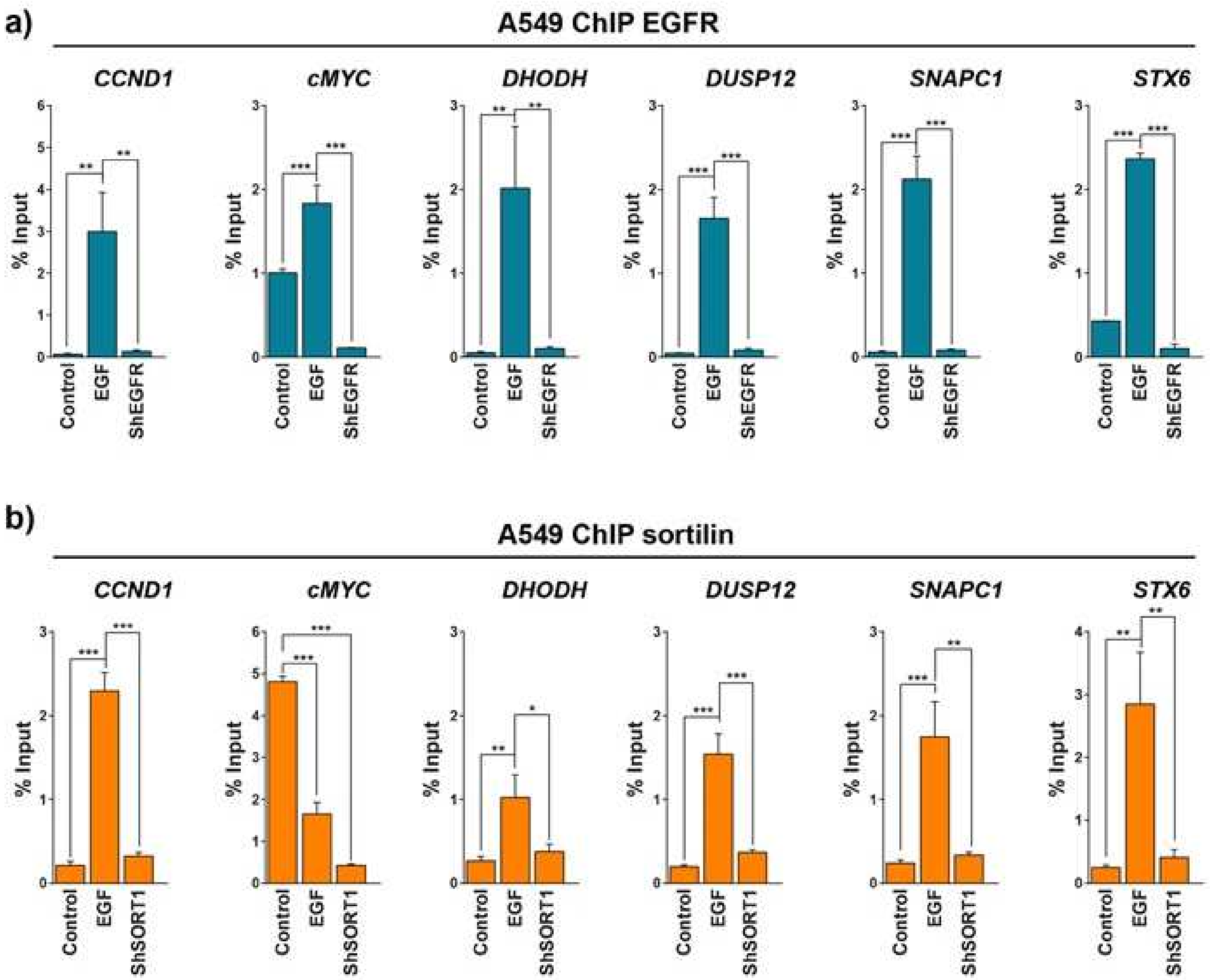

Supplementary Figure 3: EGF-associated increases in sortilin binding to DNA in A549 cells. EGFR or sortilin was immunoprecipitated, and qPCR targeting *CCND1*, *cMYC*, *DHODH*, *DUSP12*, *SNPAC1*, and *STX6* sequences was performed. **(a)** ChIP-qPCR assays of A549 cells in the absence of EGF (control) or in the presence of 50 ng/mL EGF (EGF) and of shEGFR cells (ShEGFR) in the presence of 50 ng/mL EGF. qPCR results were normalized relative to non-relevant antibodies and input. **(b)** ChIP-qPCR assays after sortilin ChIP of A549 cells without EGF (control) or in the presence of 50 ng/mL EGF (EGF), and of ShSORT1 cells (ShSORT1) in the presence of 50 ng/mL EGF. All values represent means ± SD; *p<0.05, **p<0.01, and ***p<0.001.

Supplementary_Figure_4_Lapeyronnie et al.

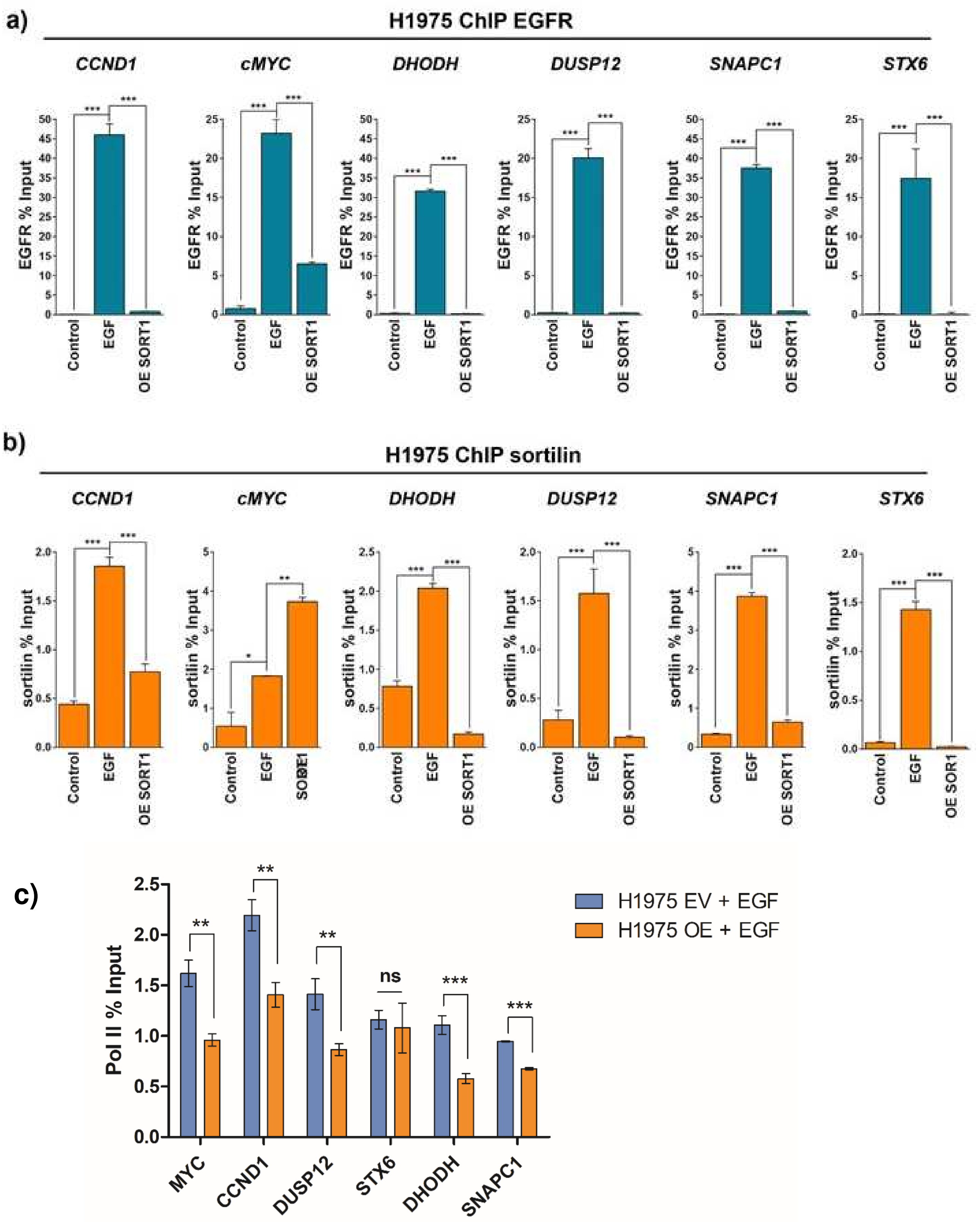

Supplementary Figure 4: Increase of sortilin binding to DNA in the presence of EGF in H1975 cells and sortilin limits Pol II binding. qPCR targeting *CCND1*, *cMYC*, *DHODH*, *DUSP12*, *SNPAC1*, and *STX6* sequences was performed after **(a)** EGFR or **(b)** sortilin ChIP. **(a-b)** ChIP-qPCR assays of H1975 cells in the absence (control) or presence of 50 ng/mL EGF (EGF) or *SORT1*

overexpressing cells (OE SORT1) in the presence of 50 ng/mL EGF. qPCR results were normalized relative to non-relevant antibodies and input. **(c)** RNA POLII ChIP assays of H1975 cells expressing empty vector (EV) and overexpressing SORT1 (OE). All values represent means ± SD; **p*<0.05, ***p*<0.01, and ****p*<0.001.

Supplementary_Figure_5_Lapeyronnie et al.

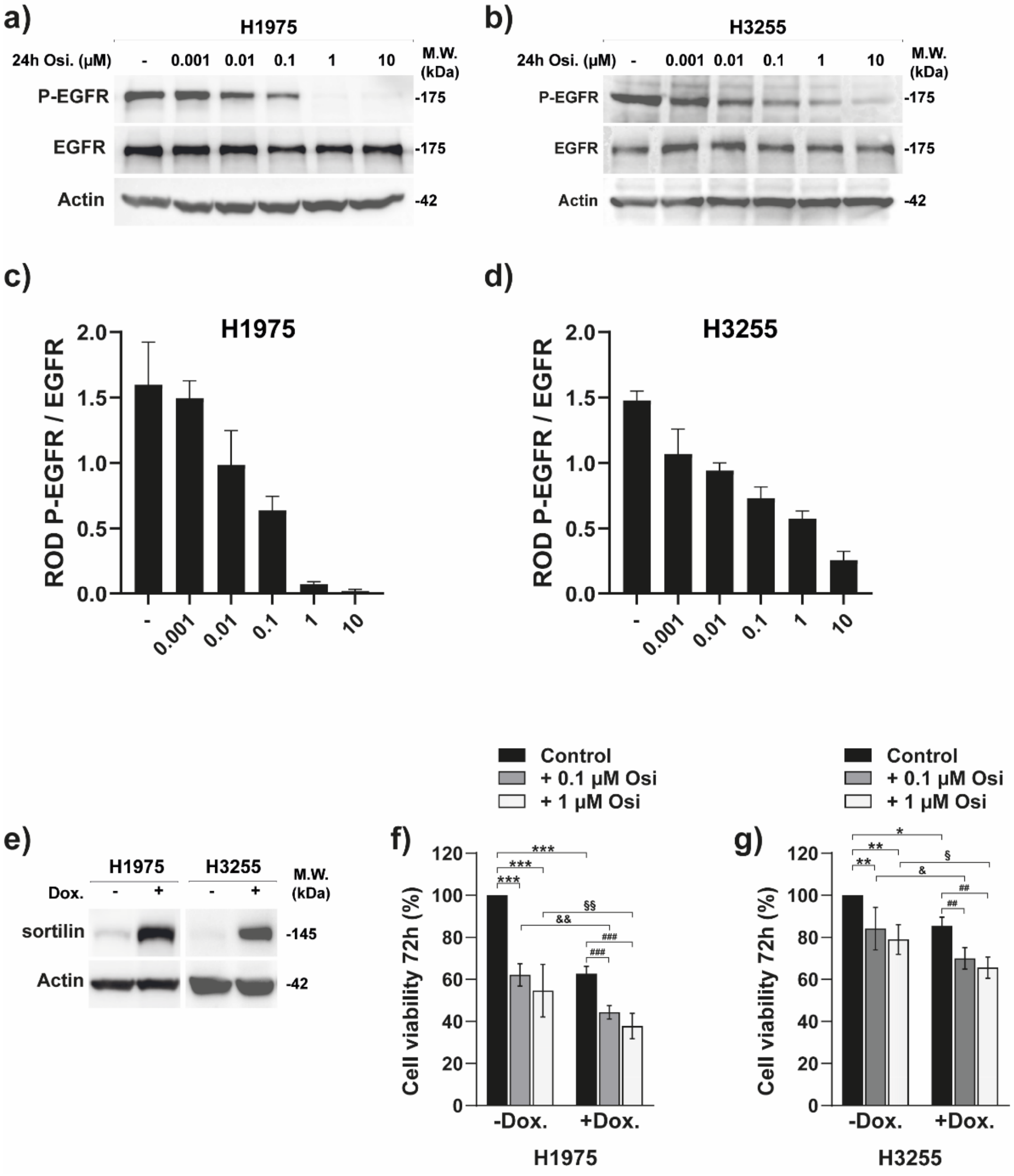

Supplementary Figure 5. Effect of osimertinib and sortilin expression on EGFR phosphorylation and cell viability in EGFR-mutant NSCLC cell lines. (a–d) H1975 and H3255 cells were treated for 24 h with increasing concentrations of osimertinib (Osi; 0.001 to 10 µM). EGFR phosphorylation levels (P-EGFR) were analyzed by immunoblotting and normalized to total EGFR expression. Actin was used as a loading control. Quantification of Relative Optic Density (ROD) of P-EGFR/EGFR ratios is shown in **(c)** for H1975 and **(d)** for H3255 cells (mean ± SD, n = 3). **(e–g)** Sortilin overexpression was induced by doxycycline (Dox; 100 nM) for 24 h prior to osimertinib exposure (0.1 or 1 µM). Sortilin expression was confirmed by immunoblotting **(e)**. Cell viability was assessed after 72 h of treatment using XTT assay **(f–g)**. Data represent mean ± SD. All values represent means ± SD; $^{*}p < 0.05$, $^{**}p < 0.01$, $^{***}p < 0.001$, $^{§}p < 0.05$, $^{§§}p < 0.01$, $^{\&\&}p < 0.01$, $^{\#\#}p < 0.01$, $^{\#\#\#}p < 0.001$.

Supplementary_Figure_6_Lapeyronnie et al.

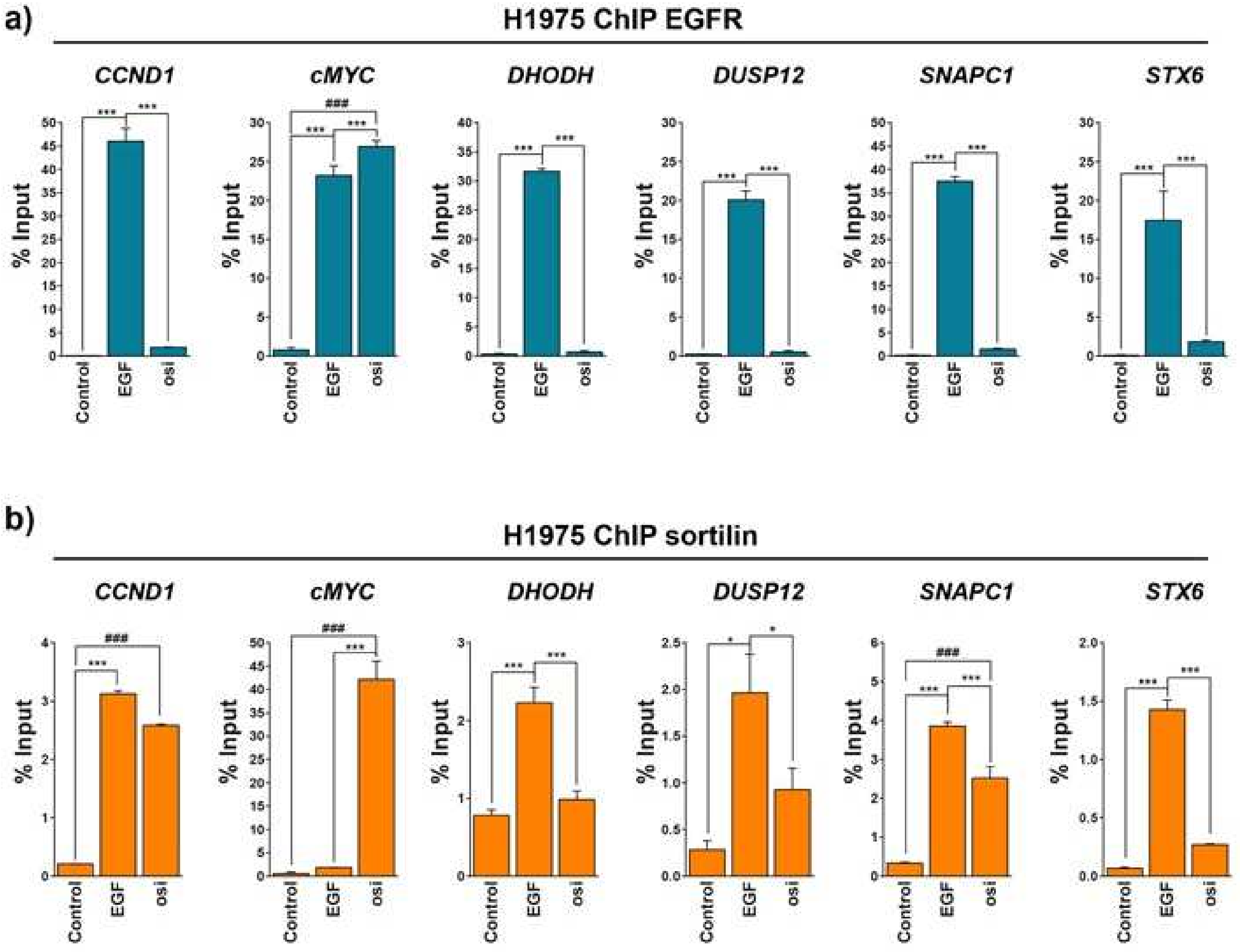

Supplementary Figure 6: Osimertinib increases EGFR and sortilin binding to DNA in H1975 cells. qPCRs amplifications targeting *CCND1*, *cMYC*, *DHODH*, *DUSP12*, *SNPAC1*, and *STX6* sequences after (a) EGFR or (b) sortilin ChIP. (a-b) ChIP-qPCR assays of H1975 cells in the absence (control) or presence (EGF) of 50 ng/mL EGF, or in the presence of 1 µM osimertinib (osi). qPCR results were normalized relative to non-relevant antibodies and input. All values represent means ± SD; *p<0.05, **p<0.01, and ***p<0.001.
